# Effects and biological consequences of the predator-mediated apparent competition I: ODE models

**DOI:** 10.1007/s00285-025-02286-x

**Published:** 2025-09-24

**Authors:** Yuan Lou, Weirun Tao, Zhi-An Wang

**Affiliations:** 1 School of Mathematical Sciences, Shanghai Jiao Tong University, Shanghai, 200240 China; 2 School of Mathematics, Southeast University, Nanjing, 211189 China; 3 Department of Applied Mathematics, The Hong Kong Polytechnic University, Hung Hom, Kowloon Hong Kong

**Keywords:** Apparent competition, Invasion, Functional response, Global stability, Coexistence and extinction, 34D05, 34D23, 92-10, 92D25

## Abstract

Predator-mediated apparent competition is an indirect negative interaction between two prey species mediated by a shared predator, which can lead to changes in population dynamics, competition outcomes and community structures. This paper is devoted to investigating the effects and biological consequences of the predator-mediated apparent competition based on a two prey species (one is native and the other is invasive) and one predator model with Holling type I and II functional responses. Through the analytical results and case studies alongside numerical simulations, we find that the initial mass of the invasive prey species, capture rates of prey species, and the predator mortality rate are all important factors determining the success/failure of invasions and the species coexistence/extinction. The global dynamics can be completely classified for the Holling type I functional response, but can only be partially determined for the Holling type II functional response. For the Holling type I functional response, we find that whether the invasive prey species can successfully invade to induce the predator-mediated apparent competition is entirely determined by the capture rates of prey species. For the Holling type II functional response, the dynamics are more complicated. First, if two prey species have the same ecological characteristics, then the initial mass of the invasive prey species is the key factor determining the success/failure of the invasion and hence the effect of the predator-mediated apparent competition. Whereas if two prey species have different ecological characteristics, say different capture rates, then the success of the invasion no longer depends on the initial mass of the invasive prey species, but on the capture rates. In all cases, if the invasion succeeds, then the predator-mediated apparent competition’s effectiveness essentially depends on the predator mortality rate. Precisely we show that the native prey species will die out (resp. persist) if the predator has a low (resp. moderate) mortality rate, while the predator will go extinct if it has a large mortality rate. Our study reveals that predator-mediated apparent competition is a complicated ecological process, and its effects and biological consequences depend upon many possible factors.

## Introduction

Predation is a primary determinant of the structure and function of ecological systems for maintaining biological diversity and balance (cf. Holt and Polis 1997; Schmitz 2007). This sounds like a paradoxical statement, as predators kill and consume prey, therefore seeming to cause death, not life. Indeed by doing so, predators may keep other species (like damaging pests) in check and ensure that a multitude of species occupying a variety of environmental niches can survive and thrive. For instance, without the regulation of predators, prey populations may reproduce beyond the carrying capacity of their environments, decimating the populations of smaller animals, plants, and coral reefs. As these species decline, additional organisms that rely on their presence will also decline, resulting in a domino effect that can ultimately push populations and habitats beyond the threshold of recovery. Predators can impact the ecosystem in enormously different ways, and hence gaining a comprehensive understanding of the role of predators in ecosystems is a daunting task. Nevertheless, theoretical models alongside analysis can play an important part in interpreting observed patterns/phenomena and making qualitative predictions, and in particular could pinpoint which processes, interactions, or parameter values are responsible for observed behaviors. Competition occurs at the same trophic level, while predation happens between different trophic levels. Though competition and predation can be intertwined directly or indirectly, these two ecological processes are often investigated separately in the existing research.

For the modeling of direct interspecific competition, the population growth rate of each species is described by a first-order differential equation

$$\begin{aligned} \frac{dN_i}{dt}=F_i\left( N_1, N_2, \ldots , N_i, \ldots \right) . \end{aligned}$$

The species *i* and *j* are competing if $\frac{\partial F_i}{\partial N_j}, \frac{\partial F_j}{\partial N_i}<0$ at equilibrium (cf. May 2001). Indirect interactions between two organisms are mediated or transmitted by a third one. In particular, there is a special indirect negative interaction, called “apparent competition” (cf. Holt 1977; Holt and Bonsall 2017), that happens between victim species mediated through the action of one or more species of shared natural enemies (e.g., predators, herbivores, omnivores, parasitoids, and pathogens). The apparent competition is usually denoted by $(-,-)$, which means a reciprocal negative interaction between each pair of victim species in the presence of a shared natural enemy. Moreover, there are also other types of enemy-mediated indirect interactions, including apparent mutualism $(+,+)$, apparent predation $(+,-)$, apparent commensalism $(+,0)$ and apparent amensalism $(-,0)$ (cf. Chailleux et al. 2014; Chaneton and Bonsall 2000; Holt and Bonsall 2017 and references therein).

**Fig. 1:**
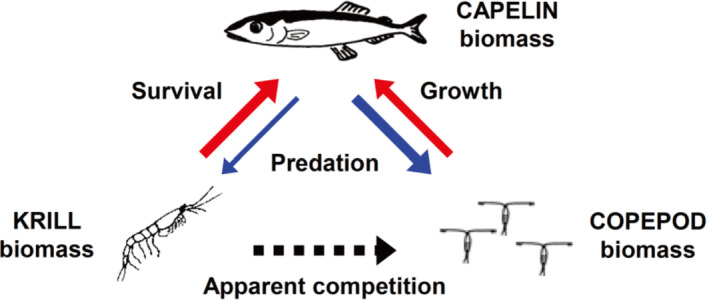
Apparent competition between krill and copepods mediated by capelin in the Barents sea. The arrow width is approximately proportional to the strength of the effect size. Bottom-up effects are shown in red, and top-down in blue. (cf. (Stige et al. 2018, Fig. 1))

In the predator-prey system with one predator and one prey, the specialist predator cannot generally take the prey to extinction as the predators usually starve to death before they can find the last prey. However, if fueled by a secondary prey species, the predator may take the native prey species to a lower level. This process is called the predator-mediated apparent competition introduced by Holt (1977) where a species indirectly and negatively affects another species that shares the same predator by influencing predator abundance of biomass. Hereafter, we shall refer this secondary prey species as an invasive prey species for convenience. It has long been recognized as a widespread phenomenon observed in many ecological communities (cf. Chaneton and Bonsall 2000; DeCesare et al. 2010). In the experiment of Karban et al. (1994), releases of Willamette mites alone, or releases of predatory mites alone, failed to reduce populations of the damaging Pacific spider mite. However, when both herbivorous Willamette mites and predatory mites were released together, populations of Pacific mites were reduced. In Stige et al. (2018), apparent competition between krill and copepods mediated by capelin in the Barents Sea (see a schematic representation in Fig. 1) was employed to advocate that a krill invasion could affect copepod biomass negatively and result in the decrease of copepod biomass. This process involves both bottom-up and top-down effects, where the bottom-up effect influences communities from lower to higher trophic levels of the food web, and the top-down effect is vice versa. However, apparent competition may be difficult to detect or measure due to its indirect nature and the potential for concurrent exploitative competition or other community effects Stige et al. (2018).

It was pointed out in Holt and Bonsall (2017) that the idea that species can engage in apparent competition by sharing a predator has a venerable history in ecology (cf. Williamson 1957 and (Lotka 1925, pp. 94-95)). The mathematical model describing predator-mediated apparent competition was first introduced by Holt Holt (1977), and can be written as the following general form for a single predator species feeding on multiple prey (see also Holt and Bonsall 2017):

1.1 $$\begin{aligned} {\left\{ \begin{array}{ll} \frac{d u_i}{d t}=F_i(\vec {u},w)=u_i\left[ g_i\left( u_i\right) -f_i(\vec {u}) w\right] ,\\ \frac{d w}{d t}=G(\vec {u},w)=w F(\vec {u}),& \\ \end{array}\right. } \end{aligned}$$

where *w* and $u_i$ are densities of the predator and prey species *i*, the arrow over *u* denotes a vector of prey abundances, $F_i$ is the total growth rate of prey species *i* and *G* is the growth rate of the predator. In the first equation of (1.1), $g_i(u_i)$ is the inherent per capita growth rate of the prey *i* in the absence of the predator, $f_i(\vec {u})$ is the functional response of the predator to prey species *i* and the quantity $f_i(\vec {u}) w$ is the per capita rate of mortality from predation experienced by prey species *i*. The right-hand side of the second equation of (1.1) states that the per capita growth rate $F(\vec {u})$ of the predator depends on prey availability. Focusing on the predator-mediated apparent competition (i.e., indirect interaction), it is assumed in (1.1) that direct interspecific competition among prey species is negligible.

Though the importance of the predator-mediated apparent competition has been extensively discussed in the biological literature (see Chaneton and Bonsall 2000; Stige et al. 2018; Karban et al. 1994; DeCesare et al. 2010 and references therein), mathematical studies on this topic are much less numerous than those for the classical predator-prey or direct competition systems (e.g. see Robert 2003; Cosner 2014; Kang and Wedekin 2013; Murdoch et al. 2013; Ni 2011; Ryan and Cantrell 2015; Sapoukhina et al. 2003; Wang et al. 2016 and references therein). Existing literature on two competing prey - one predator temporal (ODE) models has explored various scenarios. Numerical investigations in Caswell (1978) and Abrams (1999) revealed cyclic or chaotic dynamics under frequency-dependent and saturated functional responses with prey interactions, respectively. The periodic pattern was shown to exist in a two prey-one predator fast-slow dynamical system with switches of feeding between two prey species by the geometric singular perturbation method in Piltz et al. (2017). Elementary analyses in Vance (1978) gave the conditions for the existence of equilibria for frequency-dependent and Holling type I functional responses and numerically show that the predator’s presence makes competitive coexistence possible. For the Holling type I functional response, Hsu (1981) characterized equilibrium stability, while Mimura and Kan-on (1986) analytically studied spatial segregation patterns by adding random diffusions to the ODE system. We note all prior works incorporated direct competition between two prey species and relied predominantly on numerical exploration, except for the limited analytical treatments in Hsu (1981), Mimura and Kan-on (1986), Piltz et al. (2017). This paper shifts focus to the indirect competition between two prey species mediated by a shared predator. To emphasize the predation-driven indirect interactions between two prey species while enhancing analytical tractability, we exclude direct prey-prey competition to streamline model complexity. Building on equation (1.1), we thus formulate the following predator-mediated apparent competition model featuring two prey species and one shared predator:

1.2 $$\begin{aligned} {\left\{ \begin{array}{ll} u_{t}= u\left( 1-u / K_{1}\right) -w f_{1}(u), \quad & {t>0,}\\ v_{t}= v\left( 1-v / K_{2}\right) -w f_{2}(v), \quad & {t>0,}\\ w_{t}=w\left( \beta _{1} f_{1}(u)+\beta _{2} f_{2}(v)-\theta \right) , \quad & {t>0,}\\ {(u,v,w)(0)=(u_0,v_0,w_0)},& \end{array}\right. } \end{aligned}$$

where *u*(*t*), *v*(*t*) and *w*(*t*) represent the densities of the native prey species, the invasive prey species, and the shared predator species at time *t*, respectively. The initial data $u_0,v_0,w_0$ are assumed to be positive. The function $f_i(i=1,2)$ and parameters have the following biological interpretations:

- $$f_i$$, $$i=1,2$$, - functional responses;
- $K_i$, $i=1,2$, - carrying capacities for the prey species;
- $\beta _i$, $i=1,2$, - trophic efficiency (conversion rates);
- $\theta$ - mortality rate of the predator.

All the parameters shown above are positive. For definiteness, we consider two types of functional responses:

1.3 $$\begin{aligned} f_i(s)&=\alpha _i s,\qquad \quad \ i=1,2,\ (\text {Holling type I}), \end{aligned}$$

1.4 $$\begin{aligned} f_i(s)&=\frac{\gamma _i s}{1+\gamma _i h_i s},~\ i=1,2, \ (\text {Holling type II}), \end{aligned}$$

where $\alpha _i$ and $\gamma _i$, $i=1,2$, denote the capture rates (i.e., the rates at which prey species are captured), and $h_i>0$, $i=1,2$, represents the handling time.

Using rigorous analyses and quantitative computations, we investigate how system parameters and initial conditions influence the effectiveness of predator-mediated apparent competition. Since the predator-mediated apparent competition involves an introduction (or invasion) of the secondary prey species which is also a food supply to the shared predator, the invasion may not be successful and consequently, the predator-mediated apparent competition will not take effect in the long run. Therefore the first aim of this paper is to investigate

**A1**.: Under what conditions, the invasive prey species can successfully invade to promote the predator-mediated apparent competition?

If the invasive prey species invades successfully and supplies additional food to the predator, then the native prey species will be under more intensive predation pressure, possibly resulting in a population decrease or even extinction. Hence the second aim of this paper is to address

**A2**.: Whether the predator-mediated apparent competition could reduce the biomass of the native prey species or even cause the native species to go extinct? If so, what conditions are required, and which processes are the main determinants?

In this paper, we shall apply rigorous analysis along with numerical simulations to explore the above two questions. First we can fully characterize the global dynamics of (1.2) with the Holling type I functional response, proving that no non-constant patterns can emerge (see Theorem 2.1). In contrast, the Holling type II functional response can induce complex dynamics and emergent patterns. To the best of our knowledge, both our analytical and numerical findings are new, as the Holling type II functional response has not been analyzed in the literature for two prey - one predator systems. This work not only serves as a meaningful extension of existing results but also uncovers a rich landscape of dynamics such as periodic oscillations or bistability phenomenon, which are absent in the Holling type I functional response. These insights highlight the critical role of functional response forms in shaping ecological system behavior, offering new perspectives for theoretical ecology and mathematical modeling. More critically, we perform detailed qualitative and quantitative analyses to pinpoint that capture rates of prey species, the predator mortality rate and the initial mass of the invasive prey species are all possible key factors governing the effects and outcomes of predator-mediated apparent competition in regulating native prey abundance, depending on the functional responses and ecological traits of prey species as summarized in Section 4. Our results provide mechanistic insights into how predator functional responses and ecological traits of species collectively determine the success of invasive prey introduction as a tool for native prey control.

The rest of this paper is organized as follows. In Sec. 2, we state our main mathematical results on the global stability of the system (1.2) with (1.3) and (1.4), and the relevant proofs are given in Appendix A. In Sec. 3, we focus on the case of the Holling type II functional response and conduct case studies to pinpoint the main factors determining the effects and biological consequences of the predator-mediated apparent competition. In Sec. 4, we summarize our main findings and discuss several open questions.

## Global stability results

This section outlines our primary mathematical findings. We first introduce some notation used throughout the paper and then proceed to state the main results. Let

$$\begin{aligned} \begin{array}{ll} L_i:=\beta _if_i(K_i),\qquad & \lambda _i:=\frac{1}{\gamma _ih_i},\quad i=1,2,\\ L :=L_1+L_2,\quad & \theta _0:=\max \left\{ (1-\frac{\alpha _1}{\alpha _2})L_1, (1-\frac{\alpha _2}{\alpha _1})L_2 \right\} . \end{array} \end{aligned}$$

We denote the equilibrium of (1.2) by $E_s=\left( u_s,v_s,w_s\right)$, which includes the extinction equilibrium, predator-free equilibrium, semi-coexistence equilibrium and coexistence equilibrium listed in Table 1, where the coexistence equilibrium $E_*=(u_*,v_*,w_*)$ is obtained by solving (1.2) for $u,v,w>0$. To differentiate coexistence equilibria for different functional responses, we utilize the notation

$$\begin{aligned} E_*= {\left\{ \begin{array}{ll} P_*,\qquad & \text {if }(1.3)\text { holds},\\ Q_*,& \text {if }(1.4)\text { holds}. \end{array}\right. } \end{aligned}$$

Moreover, in the case of the Holling type I functional response (1.3), the coexistence equilibrium $P_*$ is uniquely given by

$$\begin{aligned} P_*=\left( \frac{K_{1} \left[ (\alpha _2-\alpha _1)L_2+\alpha _1\theta \right] }{\alpha _1L_1+\alpha _2L_2}, \frac{K_{2} \left[ (\alpha _1-\alpha _2)L_1+\alpha _2\theta \right] }{\alpha _1L_1+\alpha _2L_2}, \frac{L-\theta }{\alpha _1L_1+\alpha _2L_2}\right) , \end{aligned}$$

while in the case of the Holling type II functional response (1.4), the coexistence equilibrium $Q_*$ may not exist, may exist and be unique, or may exist but not be unique (see Remark 2.1).

### Remark 2.1

For the system (1.2) with the Holling type II functional response (1.4), it is difficult to find the necessary and sufficient conditions for the existence of $Q_*$ for general system parameters. Note that $0<\theta <L$ is a necessary but not sufficient condition for the existence of $Q_*$. Indeed, the necessity is apparent since it is easy to see that $u_*<K_1$, $v_*<K_2$, and thus

$$\begin{aligned} \theta =\beta _{1} f_{1}(u_*)+\beta _{2} f_{2}(v_*)<\beta _{1} f_{1}(K_1)+\beta _{2} f_{2}(K_2)=L, \end{aligned}$$

where we have used the fact that $f_i(s)$, $i=1,2$, strictly increases with respect to $s>0$. However, if

$$\begin{aligned} \theta =\frac{3}{5},\ K_1=2,\ K_2=3, \quad \text {and}\quad \beta _i=\gamma _i=h_i=1,\ i=1,2, \end{aligned}$$

then the system (1.2) with (1.4) has no coexistence equilibria though ${0<}\theta <L=\frac{17}{12}$.

Clearly we have $$L_1,L_2,L>0$$, $$0\le \theta _0<L$$ and $$\theta _0=0$$ if and only if $$\alpha _1=\alpha _2$$. For the global stability of equilibria of systems (1.2), it is easy to find that the equilibria $$E_0$$, $$E_u $$, $$E_v $$ are saddles for $$\theta >0$$, and $$E_{uv}$$ is also a saddle for $$\theta \in (0,L)$$ (see Lemma 3.1). Therefore, we will focus on analyzing the global stability of the equilibrium $$E_{uv}$$ for $$\theta \ge L$$, and the semi-coexistence/coexistence equilibria for $$\theta <L$$. Now we can state our main results.

### Theorem 2.1

(Global stability for Holling type I). Let $f_1(u)$ and $f_2(v)$ be given by (1.3). Then the following global stability results hold for (1.2).

(i) If $\alpha _1<\alpha _2$ (resp. $\alpha _1>\alpha _2$) and $\theta \in (0,\theta _0]$, then the semi-coexistence equilibrium $P_1$ (resp. $P_2$) is globally asymptotically stable.
(ii) If $\theta \in (\theta _0,L )$, then the unique coexistence equilibrium $P_*=\left( u_*,v_*,w_*\right)$ of (1.2) is globally asymptotically stable.
(iii) If $\theta \ge L$, then the equilibrium $E_{uv}$ is globally asymptotically stable.

**Table 1:** Equilibria of the system (1.2) with (1.3) or (1.4)

Type of equilibria		Expression of equilibria	Necessary and sufficient condition
Extinction equilibria		$E_0=(0,0,0)$	$\theta >0$
Predator-free equilibria		$E_u=(K_1,0,0),\ E_v=(0,K_2,0),\ E_{uv}=(K_1,K_2,0)$	$\theta >0$
Semi-coexistence equilibria	(1.3)	$P_1=\left( u_{P_1},0,w_{P_1}\right) =\left( \frac{\theta }{\alpha _1\beta _1}, 0,\frac{L_1-\theta }{\alpha _1L_1}\right)$	$0<\theta <L_1$
		$P_2=\left( 0,v_{P_2},w_{P_2}\right) =\left( 0,\frac{\theta }{\alpha _2\beta _2},\frac{L_2-\theta }{\alpha _2L_2}\right)$	$0<\theta <L_2$
	(1.4)	$Q_1=\left( u_{Q_1},0,w_{Q_1}\right) =\left( \frac{\theta }{(\beta _1 -h_1 \theta )\gamma _1}, 0, \frac{\beta _1 \left( L_1 -\theta \right) }{ \gamma _1f_1(K_1)(\beta _1 - h_1 \theta )^2}\right)$	$0<\theta <L_1$
		$Q_2=\left( 0,v_{Q_2},w_{Q_2}\right) =\left( 0, \frac{\theta }{ (\beta _2 - h_2 \theta )\gamma _2}, \frac{\beta _2 \left( L_2-\theta \right) }{ \gamma _2 f_2(K_2) (\beta _2 - h_2 \theta )^2}\right)$	$0<\theta <L_2$
Coexistence equilibria	(1.3)	$P_*$	$\theta _0<\theta <L$
	(1.4)	$Q_*$	Unclear (see Remark 2.1)

### Theorem 2.2

(Global stability for Holling type II). Let $f_1(u)$ and $f_2(v)$ be given by (1.4). Then the following global stability results hold for (1.2).

(i) Let $\theta \in (0,L_1)$. Then the semi-coexistence equilibrium $Q_1$ is globally asymptotically stable if
  2.1 $$\begin{aligned} (K_1,K_2)\in \Lambda _1:=\left\{ (K_1,K_2)\ \bigg |\ K_1\le \lambda _1+u_{Q_1},\ \frac{K_2}{f_2(K_2)}\le w_{Q_1}\right\} , \end{aligned}$$
  where “=” in $\frac{K_2}{f_2(K_2)}\le w_{Q_1}$ holds only in the case of $v_0\le K_2$.
(ii) Let $\theta \in (0,L_2)$. Then the semi-coexistence equilibrium $Q_2$ is globally asymptotically stable if
  2.2 $$\begin{aligned} (K_1,K_2)\in \Lambda _2:=\left\{ (K_1,K_2)\ \bigg |\ K_2\le \lambda _2+v_{Q_2},\ \frac{K_1}{f_1(K_1)}\le w_{Q_2}\right\} , \end{aligned}$$
  where “=” in $\frac{K_1}{f_1(K_1)}\le w_{Q_2}$ holds only in the case of $u_0\le K_1$.
(iii) Let $\theta \in (0,L)$ and coexistence equilibrium $Q_*=\left( u_*,v_*,w_*\right)$ exist. Then $Q_*$ is globally asymptotically stable if
  2.3 $$\begin{aligned} (K_1,K_2)\in \Lambda _*:=\left\{ (K_1,K_2)\ \bigg |\ K_1\le \lambda _1+u_*,\ K_2\le \lambda _2+v_*\right\} . \end{aligned}$$
(iv) Let $\theta \ge L$. Then the equilibrium $E_{uv}$ is globally asymptotically stable.

### Remark 2.2

We note that the sets $\Lambda _1$, $\Lambda _2$ and ${\Lambda _*}$ given in (2.1)-(2.3) are mutually disjoint. See Appendix B for the detailed proof.

### Remark 2.3

In view of Theorem 2.1, the global stability of the system (1.2) with Holling type I functional response (1.3) can be completely classified, as summarized in Table 2. However, for the Holling type II functional response (1.4), there are some gaps (see Table 3) left in the global stability for $0<\theta <L$.

**Table 2:** Global stability of equilibria of the system (1.2) with (1.3)

	$\theta \in (0,\theta _0]$	$\theta \in (\theta _0,L)$	$\theta \in [L,\infty )$
$\alpha _1>\alpha _2$	$P_2$ is GAS	$P_*$ is GAS	$E_{uv}$ is GAS
$\alpha _1<\alpha _2$	$P_1$ is GAS	$P_*$ is GAS	$E_{uv}$ is GAS
$\alpha _1=\alpha _2\ (\Longleftrightarrow \theta _0=0)$		$P_*$ is GAS	$E_{uv}$ is GAS

*Note*: Here the notations “GAS” and “$\Longleftrightarrow$” denote “globally asymptotically stable” and “if and only if”, respectively

**Table 3:** Global stability of equilibria of the system (1.2) with (1.4)

$i\in \left\{ 1,2\right\}$	$\theta \in (0,L_i)$	$\theta \in [L_i,L)$	$\theta \in [L,\infty )$
$(K_1,K_2)\in \Lambda _i$	$Q_i$ is GAS	Unclear	$E_{uv}$ is GAS
$(K_1,K_2)\in \Lambda _*$	$Q_*$ is GAS	$Q_*$ is GAS	$E_{uv}$ is GAS
$(K_1,K_2)\not \in \Lambda _1\cup \Lambda _2\cup \Lambda _*$	Unclear	Unclear	$E_{uv}$ is GAS

*Note*: Here the notation “GAS” has the same interpretation as in Table 2

Theorem 2.1 and Theorem 2.2 will be proved by the Lyapunov function method along with LaSalle’s invariant principle. The proofs are given in Appendix A.

## Numerical simulations and biological implications

From Table 2, we see that the global stability of solutions to (1.2) with (1.3) has been completely classified and there are no gaps left for the global stability of solutions. In contrast, there are some parameter gaps in which the global dynamics of (1.2) with (1.4) remain unknown (see Table 3). In the following, we shall numerically explore the global dynamics of (1.2) with (1.4) in these gaps. It is well known that one predator and one prey models with Holling type II functional response may have stable time-periodic solutions (cf. Cheng 1981). Therefore we anticipate that periodic solutions may arise from the system (1.2) with the Holling type II functional response. We shall also investigate the effect of the predator-mediated apparent competition on the population dynamics.

The associated Jacobian matrix of the system (1.2) at an equilibrium $E_s=(u_s, v_s, w_s)$ is

$$\begin{aligned} \mathcal {J}(E_s)&= \left( \begin{array}{ccc} 1-\frac{2 u_s}{K_1}-w_s f_1'(u_s) & 0 & -f_1(u_s) \\ 0 & 1-\frac{2 v_s}{K_2}-w_s f_2'(v_s) & -f_2(v_s) \\ \beta _1 w_s f_1'(u_s)& \beta _2 w_s f_2'(v_s) & \beta _{1} f_{1}(u_s)+\beta _{2} f_{2}(v_s)-\theta \\ \end{array} \right) \\&=: \left( \begin{array}{ccc} J_{11} & 0 & J_{13} \\ 0 & J_{22} & J_{23} \\ J_{31}& J_{32}& J_{33} \\ \end{array} \right) . \end{aligned}$$

We denote the three eigenvalues of $\mathcal {J}(E_s)$ by $\rho _1$, $\rho _-$ and $\rho _+$, which are the roots of

3.1 $$\begin{aligned} \rho ^3+a_2\rho ^2+a_1\rho +a_0=0, \end{aligned}$$

where $a_i=a_i(E_s)$, $i=0,1,2$, are given by

$$\begin{aligned} \left\{ \begin{array}{llll} a_0:= J_{11} J_{22} J_{33}-J_{11} J_{23} J_{32}-J_{13} J_{22} J_{31},\\ a_1:=J_{11} J_{22}+J_{11} J_{33}+J_{22} J_{33}-J_{13} J_{31}-J_{23} J_{32},\\ a_2:=-(J_{11}+J_{22}+J_{33}). \end{array} \right. \end{aligned}$$

It follows from the Routh-Hurwitz criterion (cf. (Murray 2002, Appendix B)) that all roots of (3.1) have negative real parts if and only if

$$\begin{aligned} a_0,a_1,a_2>0 \quad \text {and}\quad a_1a_2-a_0>0. \end{aligned}$$

Next, we use the above results to study the stability of all equilibria. First from Theorem 2.2 (iv) it follows that $E_{uv}$ is globally asymptotically stable for $\theta \ge L$. The following results can also be easily obtained.

### Lemma 3.1

The equilibria $$E_0$$, $$E_u $$, $$E_v $$ are saddles for any $$\theta >0$$. The equilibrium $$E_{uv}$$ is a saddle for $$\theta \in (0,L )$$, while $$E_{uv}$$ is globally asymptotically stable for $$\theta \ge L$$.

### Proof

With simple calculations, one can easily find that the eigenvalues of $\mathcal {J}$ at the four equilibria $E_0, E_u, E_v, E_{uv}$ are

$$\begin{aligned} \left\{ \begin{array}{lll} \rho _1= -\theta ,& \rho _\pm = 1,& \text {if }E_s=E_0,\\ \rho _1= L_1-\theta ,\qquad & \rho _\pm = \pm 1,\qquad \qquad & \text {if }E_s=E_u,\\ \rho _1= L_2-\theta ,& \rho _\pm = \pm 1,\quad & \text {if }E_s=E_v,\\ \rho _1= L -\theta ,\quad & \rho _\pm = -1,\quad & \text {if }E_s=E_{uv}, \end{array} \right. \end{aligned}$$

which completes the proof. $\square$

We next investigate the stability of the semi-coexistence equilibria $Q_1$, $Q_2$, and coexistence equilibrium $Q_*$. It turns out that the stability analysis for these equilibria of (1.2) with Holling type II functional response (1.4) is too complicated for explicit stability/instability conditions. For clarity and definiteness, we assume that the handling time for the two prey species is the same by simply letting $h_1=h_2=1$. By (1.4), it holds that

3.2 $$\begin{aligned} f_i(s)=\frac{s}{\frac{1}{\gamma _i}+ s}=:\frac{s}{\lambda _i+ s}, \,\quad s\ge 0,\ i=1,2. \end{aligned}$$

In what follows, we shall use (3.2) instead of (1.4) as the Holling type II functional response to undertake case studies along with numerical simulations. As illustrated in (Holt and Bonsall 2017, Figure 1), predator-mediated apparent competition among two prey species may be symmetric or asymmetric. Hence we shall distinguish these two scenarios in our subsequent analysis.

- **Symmetric apparent competition**: The two prey species have the same ecological characteristics, namely they are different phenotypes of the same species. In this case, we will consider
  $$\begin{aligned} K_i=K,\ \beta _i=\beta ,\ \gamma _i=\gamma ,\ h_i=h,\quad i=1,2, \end{aligned}$$
  where $K,\beta ,\gamma$ and *h* are positive constants.
- **Asymmetric apparent competition**: The prey species have different ecological characteristics. Such prey species may be dissimilar in many ways, such as the carrying capacity, trophic efficiency, the rate of being captured by the predator (i.e., capture rate), and so on. In this case, we may assume that the two prey species have different values for one parameter and the same values for other parameters.

### Symmetric apparent competition

For definiteness and simplicity of computations, without loss of generality, we take

3.3 $$\begin{aligned} K_1=K_2=3 \quad \text {and}\quad \beta _1=\beta _2=\lambda _1=\lambda _2=1. \end{aligned}$$

We deduce from (3.3) that $L_1=L_2=\frac{3}{4}$ and $L=\frac{3}{2}$. In addition to the equilibria $E_0$, $E_u$, $E_v$ and $E_{uv}$ of (1.2) which exist for any $\theta >0$, there are two semi-coexistence equilibria

3.4 $$\begin{aligned} {\left\{ \begin{array}{ll} Q_1=\left( \frac{ \theta }{1-\theta },0,\frac{3-4\theta }{3 (1-\theta )^2}\right) ,\\ Q_2=\left( 0,\frac{ \theta }{1-\theta },\frac{3-4\theta }{3 (1-\theta )^2}\right) , \end{array}\right. } \quad \ \text {if} \ \theta \in \left( 0,\frac{3}{4}\right) . \end{aligned}$$

With tedious but elementary calculations, one can find that there is no coexistence equilibrium if $\theta \ge \frac{3}{2}$, a unique coexistence equilibrium ${Q_*^0}$ exists if $\theta \in (0,\frac{2}{3}]\cup [1,\frac{3}{2})$ and three coexistence equilibria $Q_*^i$ (${i=0,1,2}$) exist if $\theta \in (\frac{2}{3},1)$, where

3.5 $$\begin{aligned} {\left\{ \begin{array}{ll} {Q_*^0}:=\left( \frac{\theta }{2-\theta },\frac{\theta }{2-\theta },\frac{4 (3-2 \theta )}{3 (2-\theta )^2}\right) ,\\ {Q_*^1}:=\left( 1+2 \sqrt{\frac{1-\theta }{2-\theta }},1-2 \sqrt{\frac{1-\theta }{2-\theta }},\frac{4}{3(2-\theta )}\right) ,\\ {Q_*^2}:=\left( 1-2 \sqrt{\frac{1-\theta }{2-\theta }},1+2 \sqrt{\frac{1-\theta }{2-\theta }},\frac{4}{3(2-\theta )}\right) . \end{array}\right. } \end{aligned}$$

#### Remark 3.1

In addition to the global stability result for $E_{uv}$ stated in Lemma 3.1, we can also apply Theorem 2.2 (iii) to see that ${Q_*^0}$ is globally asymptotically stable for $\theta \in [\frac{4}{3},\frac{3}{2})$ since $u_*=v_*=\frac{\theta }{2-\theta }\ge 2=K_i-\lambda _i$ ($i=1,2$).

In view of Lemma 3.1 and Remark 3.1, it remains to consider the stabilities of semi-coexistence and coexistence equilibria for $\theta \in (0,\frac{3}{2})$. We begin with the local stability of the semi-coexistence equilibria $Q_1$ and $Q_2$ for $\theta \in (0,\frac{3}{4})$.

#### Lemma 3.2

Let (3.3) hold and $\theta \in (0,\frac{3}{4})$. Then $Q_i$ ($i=1,2$) has the following properties.

- If $\theta \in \{\frac{1}{2},\frac{2}{3}\}$, then $Q_i$ ($i=1,2$) is marginally stable, where $\rho _1=-\frac{1}{3}, \rho _\pm =\pm \frac{i}{\sqrt{6}}$ if $\theta =\frac{1}{2}$, and $\rho _1=0, \rho _\pm =\frac{-2\pm \sqrt{2} i}{9}$ if $\theta =\frac{2}{3}$.
- If $\theta \in (0,\frac{1}{2})$, then $Q_i$ is a saddle-focus, where $\rho _1<0$, and $\rho _\pm$ are a pair of complex-conjugate eigenvalues with $\textrm{Re}(\rho _\pm )>0$ and ${\textrm{Im}} (\rho _\pm )\ne 0$.
- If $\theta \in (\frac{1}{2},\frac{2}{3})$, then $Q_i$ is a stable focus-node, where $\rho _1<0$, and $\rho _\pm$ are a pair of complex-conjugate eigenvalues with $\textrm{Re}(\rho _\pm )<0$ and ${\textrm{Im}} (\rho _\pm )\ne 0$.
- If $\theta \in (\frac{2}{3},\theta _1)$, then $Q_i$ is a saddle-focus, where $\rho _1>0$, and $\rho _\pm$ are a pair of complex-conjugate eigenvalues with $\textrm{Re}(\rho _\pm )<0$ and ${\textrm{Im}} (\rho _\pm )\ne 0$.
- If $\theta \in [\theta _1,\frac{3}{4})$, then $Q_i$ is a saddle with $\rho _1>0$ and $\rho _\pm <0$.

Here, $\theta _1\approx 0.6793$ is the unique real root of the equation $16 \theta ^3-37 \theta ^2+31 \theta -9=0$ for $\theta \in (0,\frac{3}{4})$.

#### Proof

We omit the proofs for brevity as they are elementary. $\square$

We next give the local stability of the coexistence equilibria.

#### Lemma 3.3

Let (3.3) hold and $\theta \in (0,\frac{3}{2})$. Then ${Q_*^0}$ has the following properties.

- If $\theta =1$, then ${Q_*^0}$ is marginally stable with $\rho _1=0$ and $\rho _\pm =\pm \frac{i}{\sqrt{3}}$.
- If $\theta \in (0,1)$, then $\rho _1>0$, and $\rho _\pm$ are a pair of complex-conjugate eigenvalues with $\mathrm Re(\rho _\pm )>0$ and ${\textrm{Im}} (\rho _\pm )\ne 0$. Therefore, ${Q_*^0}$ is an unstable focus-node.
- If $\theta \in (1,\frac{3}{4})$, then $\rho _1<0$, and $\rho _\pm$ are a pair of complex-conjugate eigenvalues with $\textrm{Re}(\rho _\pm )<0$ and ${\textrm{Im}} (\rho _\pm )\ne 0$. As a result, ${Q_*^0}$ is a stable focus-node.
- If $\theta \in [\frac{3}{4},\frac{2}{3})$, then ${Q_*^0}$ is globally asymptotically stable.

#### Proof

The proofs of the first two conclusions are omitted for brevity since they are standard and elementary. The third conclusion is a direct consequence of Theorem 2.2 (iii), see Remark 3.1. $\square$

With some tedious calculations, we also obtain the following result.

#### Lemma 3.4

Let (3.3) hold and $\theta \in (\frac{2}{3},1)$. Then $\rho _1<0$, and $\rho _\pm$ are a pair of complex-conjugate eigenvalues with $\textrm{Re}(\rho _\pm )<0$ and ${\textrm{Im}} (\rho _\pm )\ne 0$. Hence ${Q_*^1}$ and ${Q_*^2}$ are stable focus-nodes.

**Table 4:** The stability of equilibria of system (1.2) with (3.3)

Equilibria	$\theta$										
	$(0,\frac{1}{2})$	$\frac{1}{2}$	$(\frac{1}{2},\frac{2}{3})$	$\frac{2}{3}$	$(\frac{2}{3},\theta _1)$	$[\theta _1,\frac{3}{4})$	$[\frac{3}{4},1)$	1	$(1,\frac{4}{3})$	$[\frac{4}{3},\frac{3}{2})$	$[\frac{3}{2},\infty )$
$$E_0, E_u, E_v $$	Saddle	Saddle	Saddle	Saddle	Saddle	Saddle	Saddle	Saddle	Saddle	Saddle	Saddle
$E_{uv}$	Saddle	Saddle	Saddle	Saddle	Saddle	Saddle	Saddle	Saddle	Saddle	Saddle	GAS
$Q_1$, $Q_2$	SF	MS	S-FN	MS	SF	Saddle	/	/	/	/	/
${Q_*^0}$	U-FN	U-FN	U-FN	U-FN	U-FN	U-FN	U-FN	MS	S-FN	GAS	/
${Q_*^1},{Q_*^2}$	/	/	/	/	S-FN	S-FN	S-FN	/	/	/	/

Note: The abbreviations “MS”, “SF”, “S-FN”, and “U-FN” stand for “marginally stable”, “saddle-focus”, “stable focus node”, and “unstable focus node”, respectively. The notation “GAS” has the same interpretation as in Table 2. The notation “/” denotes “equilibria do not exist” and $\theta _1\approx 0.6793$ is given in Lemma 3.1

**Fig. 2:**
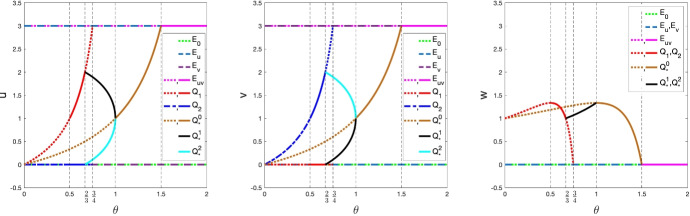
Bifurcation diagrams of system (1.2) with (3.3) versus $\theta$. The solid curves denote linearly stable equilibria, and other types of curves represent unstable equilibria

With the stability results given in Lemmas 3.1-3.4, we summarize the stability/instability properties of all equilibria in Table 4. The bifurcation diagrams of these equilibria are shown in Fig. 2. The results in Table 4 imply that if the predator mortality rate $\theta$ is sufficiently large ($\theta \ge \frac{3}{2}$), then the predator will die out and the two prey species coexist (i.e., $E_{uv}$ is globally asymptotically stable). If $\theta$ is suitably large (i.e., $\theta \in [\frac{4}{3},\frac{3}{2})$), then the predator will coexist with the two prey species (i.e., ${Q_*^0}$ is globally asymptotically stable). However, if $\theta$ is not large (i.e., $0<\theta <\frac{4}{3}$), the global dynamics largely remain unknown and different outcomes are expected from the local dynamics shown in Table 4. We shall use numerical simulations to foresee the possible global dynamics for $0<\theta <\frac{4}{3}$ and quantify the population size in the next subsection, and discuss the underlying biological implications. Our numerical simulations and biological discussion will focus on the questions **A1** and **A2** given in the Introduction. Therefore, we consider two classes of initial data. The first class of initial data is set as a perturbation of the invasive species free equilibrium $Q_1=(u_{Q_1}, 0, w_{Q_1})$ while keeping $u_{Q_1}$ and $w_{Q_1}$ unchanged, namely $(u_0, v_0,w_0)=(u_{Q_1}, R, w_{Q_1})$ with $R>0$ being a constant. The numerical results for such initial data can address the effect of the invasion of the invasive prey species on the dynamics of the native prey species, and further investigate under what conditions the native prey species is reduced in its population size or annihilated. The second class of initial data is set as a perturbation of the coexistence equilibrium $Q_*$, for which the numerical results can address the robustness of coexistence in the predator-mediated apparent competition.

**Numerical simulations and implications**. The numerical simulations for $\theta \in (0,\frac{4}{3})$ will be divided into three parts: $\theta \in (0,\frac{1}{2})$, $\theta \in [\frac{1}{2},\frac{3}{4})$ and $\theta \in [\frac{3}{4},\frac{4}{3})$, and in each part we take an arbitrary value of $\theta$ to conduct the numerical simulations.

**Fig. 3:**
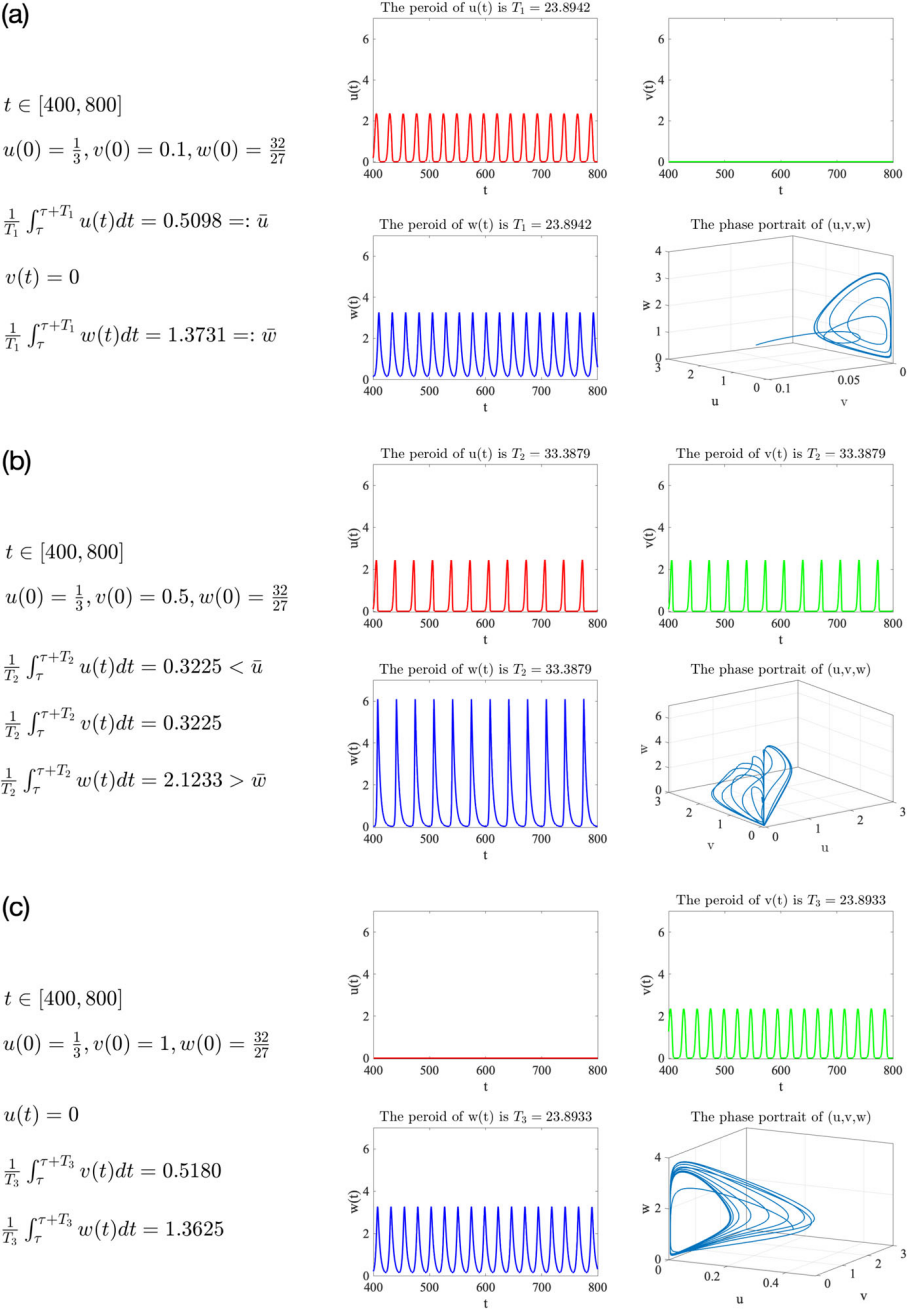
Asymptotic dynamics of the system (1.2) with (1.4) under the parameter setting (3.3) and $\theta =\frac{1}{4}$. The initial data are taken as : (a) $(\frac{1}{3},0.1,\frac{32}{27})$; (b) $(\frac{1}{3},0.5,\frac{32}{27})$; (c) $(\frac{1}{3},1,\frac{32}{27})$

**Part 1:** $\theta \in (0,\frac{1}{2})$. We take $\theta =\frac{1}{4}\in (0,\frac{1}{2})$ and focus on the semi-coexistence equilibrium $Q_1=(\frac{1}{3},0,\frac{32}{27})$ given by (3.4) which is unstable (see Table 4). The initial value is set as $(u_0, v_0, w_0)=(\frac{1}{3},R,\frac{32}{27})$ with $R>0$ denoting the initial mass of invasive prey species *v*. The numerical results for different values of *R* are plotted in Fig. 3, where we find three different typical outcomes showing that whether the invasion is successful depends on the initial biomass of invasive prey species *v* if the mortality rate of the predator is suitably small. Specifically, we have the following observations.

(i) If the initial mass $v_0$ of the invasive prey species is small (e.g. $v_0=R=0.1$), then the invasive prey species fails to invade and dies out while the native prey species coexists with the predator periodically (i.e., the solution asymptotically develops into a periodic solution $(u_1^*(t), 0, w_1^*(t))$ with period $T_1=23.8942$); see Fig. 3(a).
(ii) If the initial mass $v_0$ of the invasive prey species is medial (e.g. $v_0=R=0.5$), the invasive species *v* invades successfully and finally coexists with the native prey species *u* and the predator *w* periodically (i.e., the solution asymptotically develops into a periodic solution $(u_2^*(t), v_2^*(t), w_2^*(t))$ with period $T_2=33.3879$), but the biomass of the native prey species *u* is reduced due to the increase of the predator’s biomass, where
  $$\begin{aligned} {\left\{ \begin{array}{ll} \frac{1}{T_1} \int _0^{T_1} u_1^*(t) d t=\bar{u}=0.5098>0.3225=\frac{1}{T_2} \int _0^{T_2} u_2^*(t) d t,\\ \frac{1}{T_1} \int _0^{T_1} w_1^*(t) d t=\bar{w}=1.3625<2.1233=\frac{1}{T_2} \int _0^{T_2} w_2^*(t) d t, \end{array}\right. } \end{aligned}$$
  as shown in Fig. 3(b).
(iii) If the initial mass $$v_0$$ of the invasive prey species is large (e.g. $$v_0=R=1$$), the invasive species *v* not only invades successfully but also wipes out the native prey species via the predator-mediated apparent competition (i.e., the solution asymptotically develops into a periodic solution $$(0, v_3^*(t), w_3^*(t))$$ with period $$T_3$$ $$=23.8933)$$; see Fig. 3(c).

The above observations indicate that whether the invasive prey species can invade successfully to trigger the predator-mediated apparent competition essentially depends on the size of the initial biomass of the invasive prey species. Small initial biomass will lead to failed invasions and does not change the existing population dynamics. However, if the invasive prey species has a suitably large initial biomass, then the invasion will be successful and the predator-mediated apparent competition will take effect, resulting in the decrease or even extinction of the native prey species. To reduce the biomass of a certain species (like pests), it is suitable to employ the strategy of predator-mediated apparent competition by introducing a new (invasive) species with appropriate initial biomass.

**Fig. 4:**
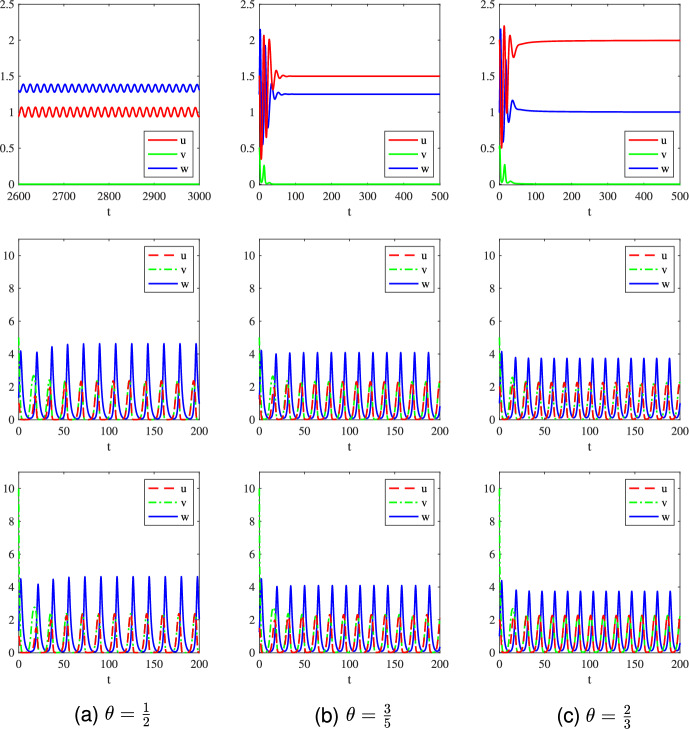
Long-time dynamics of the system (1.2) with (1.4), (3.3), and different values of $\theta \in \left\{ \frac{1}{2},\frac{3}{5},\frac{2}{3}\right\}$. The initial data are taken as $(u_0,v_0,w_0)=Q_1+(0,R,0)$, where $Q_1=(1,0,\frac{4}{3})$ in (a), $Q_1=(\frac{3}{2},0,\frac{5}{4})$ in (b), and $Q_1=(2,0,1)$ in (c); $R=0.5$ in the first row, $R=5$ in the second row, and $R=10$ in the third row

**Fig. 5:**
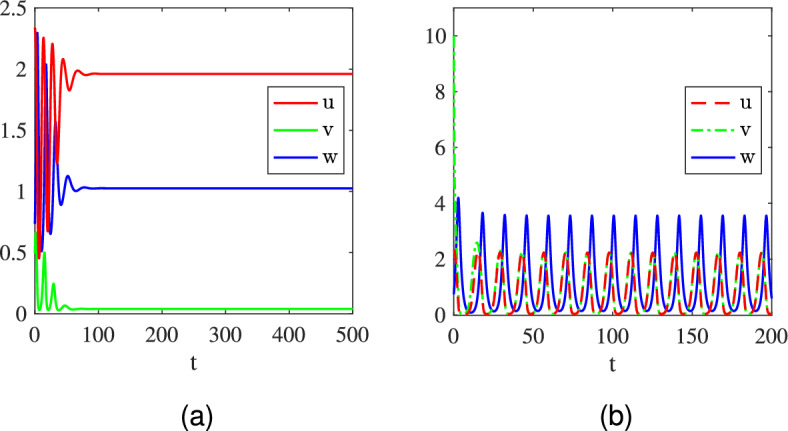
Long-time dynamics of the system (1.2) with (1.4) and parameters given in (3.3) for $\theta =\frac{7}{10}$. The initial data are taken as $(u_0,v_0,w_0)=Q_1+(0,R,0)$, where $Q_1=(\frac{7}{3},0,\frac{20}{27})$, $R=0.5$ for (a) and $R=10$ for (b)

**Part 2:** $\theta \in [\frac{1}{2},\frac{3}{4})$. In this case, we first consider the following three values for $\theta$:

$$\begin{aligned} \theta \in \left\{ \frac{1}{2},\frac{3}{5},\frac{2}{3}\right\} , \end{aligned}$$

and corresponding numerical simulations are plotted in Fig. 4. We observe similar behaviors to those for $\theta \in (0,\frac{1}{2})$ shown in Fig. 3, where the invasive species *v* will fail to invade if its initial mass is small as illustrated in the first row of Fig. 4. However, with a large initial mass, the invasive prey species can invade successfully as shown in the second row of Fig. 4, but cannot annihilate the native prey species via the predator-mediated apparent competition. This is perhaps because the predator mortality rate $\theta$ is too large to annihilate the native species even if the invasive species can boost the food supply of the predator. This result alongside the numerical simulations shown in Fig. 3 implies that whether the native prey species will be driven to extinction via the predator-mediated apparent competition depends not only on the initial mass of the invasive species but also on the mortality rate of the predator. Further increasing the value of $\theta$ to be $\theta =\frac{7}{10} \in (\theta _1, \frac{3}{4})$, at which $Q_1=(\frac{7}{3},0,\frac{20}{27})$, we find from the numerical simulations shown in Fig. 5(a) that the invasion is successful albeit small initial population abundance of the invasive species (in comparison with those in the first row of Fig. 4). Mathematically this is because $Q_1$ is a saddle and any small perturbation of $Q_1$ will result in instability. With a large predator mortality rate, the invasive species (even with a large initial mass) cannot drive the native species to extinction (see Fig. 5(b)), similar to other large values of $\theta$ shown in the second and third rows of Fig. 4. This implies that if the predator has a large mortality rate, it can not drive the native prey species to extinction even if its food supply is boosted by the invasive prey species.

Concerning the questions raised in **A1**, the above numerical results pinpointed two key factors determining successful invasion of the invasive prey species: the initial invasive mass $v_0$ and mortality rate $\theta$ of the predator. Specifically, for a fixed mortality rate $\theta$ not large, increasing the initial invasive mass $v_0$ can lead to a successful invasion. If the mortality rate $\theta$ is large, then the predator will go extinct and the mass of the native prey species will not be affected though the invasion is successful. Conversely, for a fixed initial invasive mass that is not too small, the larger mortality rate of the predator will be beneficial to the success of the invasion. Moreover, the population abundance of the native prey species will be reduced by the predator-mediated apparent competition as shown in Fig. 3. Another interesting finding in our numerical simulations is that the asymptotic profiles of the native and invasive prey species coincide as long as the non-trivial periodic coexistence state appears (see Fig. 3 to Fig. 5). This result is not yet understood and deserves further investigation.

Next, we explore how the population abundance of native prey species changes with respect to the initial invasive mass. To this end, we take the numerical results shown in Fig. 4(b) as an example. Denote the three solutions shown in Fig. 4(b) by $(u_{R}^*,v_{R}^*,w_{R}^*)(t)$ for $\theta =\frac{3}{5}$ and $R=0.5,5,10$. Then $(u_{R}^*,v_{R}^*,w_{R}^*)(t)\mid _{R=0.5}\equiv Q_1=(\frac{3}{2},0,\frac{5}{4})$ for all $t>0$, and $(u_{R}^*,v_{R}^*,w_{R}^*)(t)$ are periodic solutions with period $T_R$ for $R=5,10$. Quantitative estimates of the total population in a period for $R=0.5,5,10$ are summarized in Table 5. We see from these results that the total mass of the native prey species decreases with respect to the initial mass of the invasive prey species, as expected.

**Table 5:** Quantitative properties of $(u_{R}^*,v_{R}^*,w_{R}^*)(t)$ for $R=0.5,5,10$

*R*	0.5	5	10
Period $T_R$	/	15.3714	15.3714
$\bar{u}=\frac{1}{T_R} \int _0^{T_R} u_{R}^*(t) d t$	$\frac{3}{2}$	0.6277	0.6275
$\bar{v}=\frac{1}{T_R} \int _0^{T_R} v_{R}^*(t) d t$	0	0.6277	0.6275
$\bar{w}=\frac{1}{T_R} \int _0^{T_R} w_{R}^*(t) d t$	$\frac{5}{4}$	1.5866	1.5844

*Remark*: Here the notation “/” means “this is not a non-constant periodic case”

**Fig. 6:**
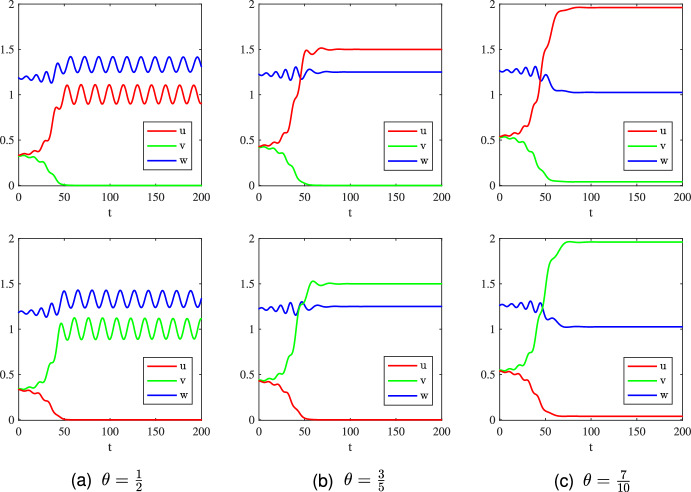
Long-time dynamics of the system (1.2) with (1.4) and parameters given in (3.3) for $\theta \in \left\{ \frac{1}{2},\frac{3}{5},\frac{7}{10}\right\}$. The initial data are taken as $(u_0,v_0,w_0)={Q_*^0}+(0,R,0)$, where $R=-0.01$ for the first row and $R=0.01$ for the second row, and ${Q_*^0}$ is given by (3.5): (a) $(\frac{1}{3},\frac{1}{3},\frac{32}{27})$; (b) $(\frac{3}{7},\frac{3}{7},\frac{60}{49})$; (c) $(\frac{7}{13},\frac{7}{13},\frac{640}{507})$

**Fig. 7:**
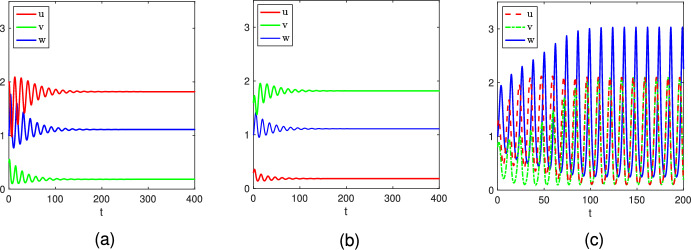
Long-time dynamics of the system (1.2) with (1.4) under the parameter setting (3.3) and $\theta =0.8$. The initial data are taken as $(u_0,v_0,w_0)$: (a) (2, 0.5, 1); (b) (0.3, 1.6, 1); (c) (1.2, 0.8, 1)

We proceed to examine whether the constant coexistence/positive solution is stable. To this end, we shall investigate the stability/instability of $Q_0^*$ which exists if $\theta <\frac{3}{2}$. The results of Theorem 2.2 show that $Q_0^*$ is globally asymptotically stable if $\theta \in [\frac{4}{3},\frac{3}{2})$. This indicates that if the mortality rate of the predator is appropriately large, then coexistence will persist as long as the invasion is successful. However, this is no longer the case if the mortality rate of the predator is suitably small, as shown in Fig. 6 where we see that any small negative (resp. positive) perturbation of one prey species density may lead to the extinction or abundance decrease of this species (resp. the other one). This indicates that the constant coexistence solution is not robust against (small) perturbations.

**Part 3:** $\theta \in [\frac{3}{4},\frac{4}{3})$. In view of Table 4, both coexistence equilibria ${Q_*^1}$ and ${Q_*^2}$ are stable for $\theta \in [\frac{3}{4},1)$, that is the system (1.2) generates bistable dynamics as illustrated in Fig. 7 for $\theta =0.8$, where

$$\begin{aligned} {Q_*^1}=\left( 1+\sqrt{\frac{2}{3}},1-\sqrt{\frac{2}{3}},\frac{10}{9}\right) , \ {Q_*^2}=\left( 1-\sqrt{\frac{2}{3}},1+\sqrt{\frac{2}{3}},\frac{10}{9}\right) . \end{aligned}$$

With an initial value $(u_0,v_0,w_0)$ which is“closer” to ${Q_*^1}$ than ${Q_*^2}$, the corresponding numerical results shown in Fig. 7(a) demonstrate that the solution converges to ${Q_*^1}$, while Fig. 7(b) illustrates the convergence of solutions to ${Q_*^2}$ when the initial value is closer to ${Q_*^2}$. We wonder if a non-constant solution may develop if the initial value is not close to either of these two stable equilibria. Hence, we choose an initial value $(u_0,v_0,w_0)=(1.2,0.5,1)$ neither close to ${Q_*^1}$ nor to ${Q_*^2}$; the corresponding numerical result shown in Fig. 7(c) demonstrates that the periodic solution will develop. But how to rigorously prove the existence of periodic solutions remains an interesting open question.

In applications, the invasive prey species may be used as a biological control agent to regulate the population size of the native prey species if they are harmful (like pests). The ideal situation is that a small number of invasive prey species can achieve this goal. The above linear stability analysis alongside numerical simulations indicates that this is unfeasible if two prey species are ecologically identical (i.e., the symmetric case). However, this is achievable when two prey species are ecologically different (i.e., asymmetric case) as to be shown in the next subsection.

### Asymmetric apparent competition

For simplicity, we first rescale the system (1.2) with (1.4). To this end, we set

3.6 $$\begin{aligned} \widetilde{u}=\frac{u}{K_1},\ \widetilde{v}=\frac{v}{K_2},\ \widetilde{w}=w,\quad (\widetilde{\gamma }_i,\widetilde{h}_i,\widetilde{\beta }_i) =(\gamma _i,h_i K_i,\beta _i K_i),\quad i=1,2. \end{aligned}$$

Substituting the above rescalings into (1.2) with (1.4) and dropping the tildes for brevity, we obtain the following rescaled system

3.7 $$\begin{aligned} {\left\{ \begin{array}{ll} u_{t}= u\left( 1-u \right) -w \frac{\gamma _1 u}{1+\gamma _1 h_1 u}, \quad & t>0,\\ v_{t}= v\left( 1-v \right) -w \frac{\gamma _2 v}{1+\gamma _2 h_2 v}, \quad & t>0,\\ w_{t}=w\left( \beta _{1} \frac{\gamma _1 u}{1+\gamma _1 h_1 u} + \beta _{2}\frac{\gamma _2 v}{1+\gamma _2 h_2 v} -\theta \right) , \quad & t>0,\\ (u,v,w)(0)=(u_0,v_0,w_0). \end{array}\right. } \end{aligned}$$

The rescaled system (3.7), which can be viewed as a special case of (3.6) with $K_1=K_2=1$, has three types of parameters: capture rates $\gamma _i$, handling times $h_i$ and conversion rates $\beta _i$, where $i=1,2$, In the following, we shall focus on the case where the two prey species have different capture rates (i.e., $\gamma _1\ne \gamma _2$), and by assuming $h_1=h_2, \beta _1=\beta _2$, we can study the effects of predator-mediated apparent competition with different capture rates. For definiteness, we set without loss of generality

3.8 $$\begin{aligned} h_i=1,\ \beta _i=b>0\ \quad \text {and}\quad 0< \gamma _2< \gamma _1=1. \end{aligned}$$

The biological meaning of parameter values set in (3.8) is that the two prey species *u* and *v* have the same handling times and conversion rates but vary in capture rates, while the predator prefers to hunt the native prey species *u* ($\gamma _1>\gamma _2$). Clearly the rescaled system (3.7) with (3.8) has four predator-free equilibria

$$\begin{aligned} E_0=(0,0,0),\ E_u=(1,0,0),\ E_v=(0,1,0),\ E_{uv}=(1,1,0),\quad \text {if }\theta >0, \end{aligned}$$

two semi-coexistence equilibria

$$\begin{aligned} {\left\{ \begin{array}{ll} Q_1=\left( u_{Q_1},0,w_{Q_1}\right) =\left( \frac{\theta }{b-\theta },0,\frac{b (b-2 \theta )}{(b-\theta )^2}\right) , \quad \  & \text {if } \theta \in \left( 0, L_1\right) ,\\ Q_2=\left( 0,v_{Q_2},w_{Q_2}\right) =\left( 0,\frac{\theta }{\gamma _2(b-\theta )},\frac{b (b \gamma _2-(1+\gamma _2) \theta )}{\gamma _2^2(b-\theta )^2}\right) , \quad \  & \text {if } \theta \in \left( 0, L_2\right) , \end{array}\right. } \end{aligned}$$

and a unique coexistence equilibrium (see Lemma C.3 in Appendix C for detailed reasons)

3.9 $$\begin{aligned} Q_*=(u_*,v_*,w_*),\quad \text {if } \theta \in (\Theta _1, L), \end{aligned}$$

where

3.10 $$\begin{aligned} {\left\{ \begin{array}{ll} L_1=\frac{b}{2}> L_2=\frac{b \gamma _2}{1+\gamma _2},\ L= L_1+ L_2<b,\\ \Theta _1=\varphi _1(\gamma _2) b\in (0, L_2),\quad \varphi _1(\gamma _2):=\frac{ \sqrt{(1-\gamma _2) (3 \gamma _2+1)}-(1-\gamma _2) (2 \gamma _2+1)}{2 \gamma _2^2}. \end{array}\right. } \end{aligned}$$

For $b>0$ and $\gamma _2\in (0,1)$, it holds that

$$\begin{aligned} {\left\{ \begin{array}{ll} \varphi _1''(\gamma _2)<0,\ \varphi _1'(\frac{2}{3})=0,\ \lim \limits _{\gamma _2\rightarrow 0}\varphi _1(\gamma _2)=\lim \limits _{\gamma _2\rightarrow 1}\varphi _1(\gamma _2)=0,\\ 0<\Theta _1\le b\varphi _1(\frac{2}{3})=\frac{b}{4}, \text { and }\Theta _1\text { attains its maximum }\frac{b}{4} \text { if and only if }\gamma _2=\frac{2}{3}. \end{array}\right. } \end{aligned}$$

This implies that $\Theta _1$ is non-monotone in $\gamma _2$, i.e., it is a convex function maximized at $\gamma _2=\frac{2}{3}$.

#### Remark 3.2

Applying Theorem 2.2 (iii)-(iv) with $K_1=K_2=1$ to system (3.7)-(3.8), we can easily find that $Q_*$ is globally asymptotically stable for $\theta \in (\Theta _1, L)$, and $E_{uv}=(1,1,0)$ is globally asymptotically stable for $\theta \ge L$. Since $\frac{1+\gamma _1 h_1}{\gamma _1}=2>\lim \limits _{\theta \rightarrow \Theta _1}w_{Q_2}=1$ for $b>0$ and $\gamma _2\in (0,1)$, the results in Theorem 2.2(ii) with $K_1=K_2=1$ are inapplicable to assert the global stability of $Q_2$ for $\theta \in (0,\Theta _1]$. However, this can be shown in the following lemma.

#### Lemma 3.5

The semi-coexistence equilibrium $Q_2$ of the rescaled system (3.7) with (3.8) is globally asymptotically stable if $\theta \in (0,\Theta _1]$.

#### Proof

Let $\theta \in (0,\Theta _1]$. Then (3.10) implies $0<\theta<\frac{b \gamma _2}{1+\gamma _2}<\frac{b}{2}$. For $t>0$, let

$$\begin{aligned} \mathcal E(t;Q_2)=bu +(b-\theta ) \left( v-v_{Q_2}-v_{Q_2}\ln \frac{v}{v_{Q_2}}\right) +\left( w-w_{Q_2}-w_{Q_2}\ln \frac{w}{w_{Q_2}}\right) . \end{aligned}$$

Then by similar arguments as in the proofs of Lemma A.2 and Lemma A.5, we have $\mathcal E (t;Q_2)>0\text { for all }(u,v,w)\ne Q_2$, and

$$\begin{aligned} \mathcal E'(t;Q_2)&= ~ b \left( 1-u -\frac{w }{1+ u}\right) u+(b-\theta )\left( 1-v - \frac{\gamma _2 w}{1+\gamma _2 v}\right) (v-v_{Q_2})\\&\quad +\left( \frac{b \gamma _2 v}{\gamma _2 v+1}+\frac{b u}{u+1}-\theta \right) (w-w_{Q_2})\\&= -(b-\theta )\frac{(1-\gamma _2+\gamma _2 (v+v_{Q_2}))}{\gamma _2 v+1}(v-v_{Q_2})^2 -\frac{b u^3}{u+1}+\frac{b u \varphi _2(\theta )}{\gamma _2^2 (u+1) (b-\theta )^2}\\&< -(b-\theta )\frac{\gamma _2 (v+v_{Q_2})}{\gamma _2 v+1}(v-v_{Q_2})^2 -\frac{b u^3}{u+1}, \end{aligned}$$

where we have used $\gamma _2\in (0,1)$ and the fact that the quadratic function

3.11 $$\begin{aligned} \varphi _2(\theta )&:=\gamma _2^2\theta ^2 +b \left( -2 \gamma _2^2+\gamma _2+1\right) \theta +b^2 (\gamma _2-1) \gamma _2\nonumber \\&=\gamma _2^2\left[ \theta +\Theta _1+b \left( \frac{1+\gamma _2}{\gamma _2^2}-2\right) \right] (\theta -\Theta _1) \end{aligned}$$

is nonpositive for $\theta \in (0,\Theta _1]$ in the last inequality. Finally, similar arguments based on the Lyapunov function method and LaSalle’s invariant principle as in the proof of Lemma A.2 complete the proof. $\square$

With Remark 3.2 and Lemma 3.5, we summarize the global stability results in Table 6 for the rescaled system (3.7) with (3.8).

**Table 6:** Global stability of (3.7) with (3.8)

$\theta$	$(0,\Theta _1]$	$(\Theta _1,L)$	$[L,\infty )$
Global stability	$Q_2\text { is GAS}$	$Q_*\text { is GAS}$	$E_{uv}\text { is GAS}$

*Note*: The notation “GAS” has the same interpretation as in Table 2. The parameter $\Theta _1$ is given in (3.10)

Under the parameter setting (3.8), the capture rate of the invasive prey species *v* is smaller than the native prey species *u*, namely $0<\gamma _2<\gamma _1=1$. According to the results shown in Table 6 for any $\theta >0$, we can derive the following biological implications.

(i) If $\theta \in (0, \Theta _1]$ (i.e., the predator has a low mortality rate), the global stability of $Q_2$ implies that the invasive prey species can invade successfully regardless of its initial population size and wipe out the native prey species via the predator-mediated apparent competition.
(ii) If $\theta \in (\Theta _1, L)$ (i.e., the predator has a moderate mortality rate), then the global stability of $Q_*$ indicates that moderate predator mortality allows the native prey species to survive and to coexist with the invasive prey species and the predator.
(iii) If $\theta \ge { L}$, the global stability of $E_{uv}$ entails that the poor physical condition of the predator (i.e., the predator has a large mortality rate) will result in the extinction of the predator even though the invasive prey species can boost the food supply to the predator.

**Fig. 8:**
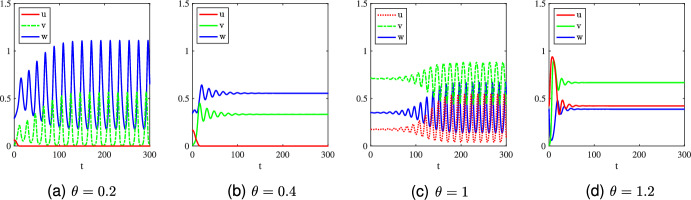
Long-time dynamics of the rescaled system (3.7) with $h_i= \beta _i=1$ ($i=1,2$), $(\gamma _1,\gamma _2)=(4,2)$ and $\theta =0.2,0.4,1,1.2$. The initial data are taken as $(u_0,v_0,w_0)=Q_1+(0,0.01,0)$ in (a)-(b), $(u_0,v_0,w_0)=Q_*+(0,0.01,0)$ in (c), and $(u_0,v_0,w_0)=(u_*,0.01,w_*)$ in (d)

The above results indicate that if the predator has a hunting preference for the native species (i.e., larger capture rate of the native prey species), then the invasive prey species can always invade successfully regardless of its initial population size. Furthermore, whether or not the native prey species can be eradicated through the predator-mediated apparent competition essentially depends upon the mortality rate of the predator (i.e., low predator mortality rate will result in the extinction of the native prey species while a moderate or large mortality rate will allow the native prey species to persist). In the general parameter set in which $0<\gamma _2<\gamma _1$, the case $0<\gamma _2<\gamma _1=1$ is only a special situation where we can completely classify the global stability of solutions as given in Table 6. For other parameter regimes contained in the set $0<\gamma _2<\gamma _1$, we can perform the linear stability analysis to obtain local stability results and employ the Lyapunov function method alongside LaSalle’s invariant principle to obtain the global stability results in partial parameter regimes, but a complete classification of global stability can not be established. Indeed, in some parameter regimes, periodic solutions may exist (see Fig. 8), and hence the global stability in the whole parameter domain is impossible. Nevertheless, the biological phenomena observed from our numerical simulations (not shown here for brevity) are essentially similar to the case $0<\gamma _2<\gamma _1=1$: the invasive prey species will always invade successfully regardless of its initial population abundance and can even wipe out the native prey species through the predator-mediated apparent competition if the mortality rate of the predator is low, while the native prey species can persist and coexist with the predator and invasive prey species if the mortality rate of the predator is moderate, where the difference from the case $0<\gamma _2<\gamma _1=1$ is that the coexistence state may be periodic or constant as shown in Fig. 8.

If we assume $0<\gamma _1<\gamma _2=1$ (i.e., the capture rate of the native prey species is smaller than that of the invasive prey species), then the results in Table 6 hold by swapping $Q_1$ with $Q_2$. This means that if the predator has a hunting preference for the invasive prey species, then a successful invasion depends heavily on the predator mortality rate (precisely, the invasion will fail for $\theta \in (0, \Theta _1]$ while succeeding for $\theta > \Theta _1$). Even if the invasion is successful, the invasive prey species is unable to wipe out the native prey species through predator-mediated apparent competition, regardless of its initial population abundance. These interesting results have significant value in applications. For instance, if we were to control the population abundance of some harmful species (like pests) by their natural enemies, we can introduce a small amount of secondary (invasive) prey species that are less preferred by their natural enemies based on the principle of predator-mediated apparent competition.

## Summary and discussion

Predator-mediated apparent competition is an indirect and negative interaction between two prey species mediated by a shared predator. As stressed in Stige et al. (2018), quantifying such indirect effects is methodologically challenging but important for understanding ecosystem function. To study the effects of predator-mediated apparent competition on population dynamics, in this paper, we propose to consider system (1.2) by viewing *u* as a native prey species and *v* as an invasive prey species, both of which share one predator *w*. We find conditions for the local and global stability of the equilibria of system (1.2) with Holling type I and II functional responses in Section 2, and employ numerical simulations to demonstrate the possible population dynamics and biological consequences due to the predator-mediated apparent competition in Section 3.

In summary, we find that if two prey species employ the Holling type I functional responses, whether the invasion is successful and hence promotes the predator-mediated apparent competition is entirely determined by their capture rates (i.e., the rates being captured by the predator). In contrast, the dynamics with the Holling type II functional responses are more complicated. First, if two prey species have the same ecological characteristics, then the initial mass of the invasive prey species is the key factor determining the success of the invasion and hence the promotion of the predator-mediated apparent competition. Whereas if two prey species have different ecological characteristics, say different capture rates without loss of generality, then the success of the invasion (i.e., the promotion of the predator-mediated apparent competition) no longer depends on the initial mass of the invasive prey species, but on the capture rates. In all cases, if the invasion succeeds, whether the native prey species can be annihilated via predator-mediated apparent competition essentially depends on the predator mortality rate (i.e., the low predator mortality rate will result in the extinction of the native prey species). These intriguing findings not only fully address the questions posed in **A1** and **A2** of Section 1 but also offer actionable insights for decision-makers when introducing alien species into ecological systems to maintain ecological balance and biodiversity.

Our present work not only pinpoints key factors promoting predator-mediated apparent competition but also shows the significant effects of predator-mediated apparent competition on the structure and stability of ecological systems. Therefore, a comprehensive understanding of the mechanism underlying dynamics of this indirect interaction is imperative. This paper only takes a (first) step forward in this direction and many interesting questions remain open.

- We consider the same functional response for both prey species, either Holling type I or Holling type II. In reality, the functional response for two prey species may be different, such as Holling type I for the native prey species and Holling type II for the invasive one, or vice versa. Then we anticipate that the dynamics might be different from those obtained in this paper. This deserves to be clarified in a future work.
- The model considered in this paper does not include spatial structure, such as random diffusion and/or directed movement (e.g. prey-taxis cf. Kareiva and Odell 1987), which are indispensable factors to make the model more realistic. This raises a natural question: what are the dynamics of the predator-mediated apparent competition with spatial structure and whether the spatial movement of species will bring significantly different effects? These interesting questions can serve as a roadmap to study spatial effects on the population dynamics of predator-mediated apparent competition and hence provide insights into the understanding of complex dynamics of ecological systems. We shall explore this question in the future.
- In the model, the direct (i.e., interference) competition of two prey species is not considered. If we include the direct competition in the model, the complexity of both qualitative and quantitative analysis will be considerably increased. However, it is still very interesting to explore how the direct competition and indirect interaction (i.e., predator-mediated apparent competition) between the two prey species jointly affect the population dynamics.

## Figures and Tables

**Fig. 9:**
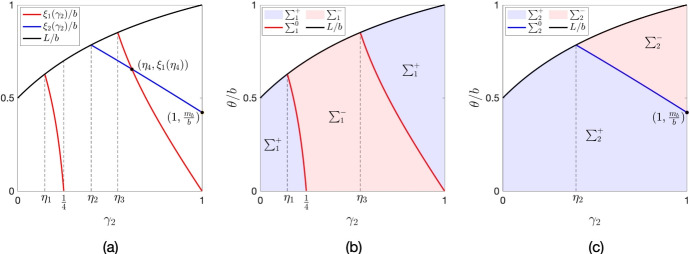
The graphs of three functions $\frac{\xi _1(\gamma _2)}{b}$, $\frac{\xi _2(\gamma _2)}{b}$ and $\frac{L}{b}$ versus $\gamma _2\in (0,1)$ are shown in **(a)**. The signs of $D_1$ and $D_2$ in the $\gamma _2$-$\theta /b$ plane within $(\gamma _2,\theta )\in (0,1)\times (0, L)$ are shown in **(b)** and **(c)**, respectively

## Data Availability

The authors declare that the manuscript has no associated data.

## Appendix A. Proof of the global stability

In this appendix, we aim to prove the global stability of the equilibria of the system (1.2). As mentioned earlier, we will primarily focus on proving the global stability of the equilibrium $E_{uv}$ for $\theta \ge L$ and semi-coexistence/coexistence equilibria for $0<\theta <L$. Before proceeding with the stability analysis, we first establish the global well-posedness of the system (1.2) by proving the following result.

### Lemma A.1

Let $(u_0, v_0, w_0) \in \mathbb {R}_{+}^3$, and let $f_1(u)$ and $f_2(v)$ be given by (1.3) or (1.4). Then the system (1.2) admits a unique nonnegative solution, which is bounded for $t{>}0$. Moreover, the solution satisfies

A1 $$\begin{aligned} \sup _{t{\geqslant }0} u(t)\le M_1,\quad \sup _{t{\geqslant }0} v(t)\le M_2,\quad \sup _{t{\geqslant }0} w(t)\le M_3, \end{aligned}$$

and

A2 $$\begin{aligned} \limsup _{t \rightarrow \infty } u(t) \le K_1,\quad \limsup _{t \rightarrow \infty } v(t) \le K_2,\quad \limsup _{t \rightarrow \infty } w(t) \le \frac{(1+\theta )^2}{4\theta }\left( \beta _1K_1+\beta _2K_2\right) , \end{aligned}$$

where the constants $M_i$, $i=1,2,3$, are given by

$$\begin{aligned} {\left\{ \begin{array}{ll} M_1:=\max \left\{ u_0,K_1\right\} ,\quad M_2:=\max \left\{ v_0,K_2\right\} ,\\ M_3:=\max \left\{ \beta _1u_0+\beta _2v_0+w_0,\frac{(1+\theta )^2}{4\theta }\left( \beta _1K_1+\beta _2K_2\right) \right\} . \end{array}\right. } \end{aligned}$$

### Proof

Since the vector field, defined by the terms on the right-hand side of the system (1.2), is smooth in $\mathbb {R}_{+}^3$, the existence theory of ordinary differential equations (cf. (Logemann and Ryan 2014, Theorem 4.18)) guarantees that the system (1.2) admits a unique maximal solution with a maximal time $T_{max}\in (0,\infty ]$. By the first equation of (1.2), we have

$$\begin{aligned} u(t)=u_0e^{\int _0^t(1-\frac{u(s)}{K_1}-\frac{w(s)f_1(s)}{u(s)})}ds\ge 0\quad \text {for all }t\in (0,T_{max}). \end{aligned}$$

We can similarly obtain $v(t),w(t)\ge 0$ for all $t\in (0,T_{max})$. Then the first equation of (1.2) shows that $u_t\le u(1-u / K_{1})$, which along with the comparison principle gives $u(t)\le \max \left\{ u_0,K_1\right\} =M_1$ for all $t\in (0,T_{max})$. Similarly, it holds that $v(t)\le \max \left\{ v_0,K_2\right\} =M_2$ for all $t\in (0,T_{max})$. Let $z(t):=\beta _1u(t)+\beta _2v(t)+w(t)$, then we have from (1.2) and Young’s inequality that

$$\begin{aligned} z_t&= ~\beta _1u\left( 1-\frac{u}{K_{1}}\right) +\beta _2 v\left( 1-\frac{v}{K_{2}}\right) -\theta \left( z-\beta _1u -\beta _2v \right) \\&= -\theta z+\beta _1\left( \left( 1+\theta \right) u-\frac{u^2}{K_1}\right) +\beta _2\left( \left( 1+\theta \right) v-\frac{v^2}{K_2}\right) \\&\le -\theta z+\frac{(1+\theta )^2}{4}\left( \beta _1K_1+\beta _2K_2\right) \quad \text {for all } t\in (0,T_{max}). \end{aligned}$$

By the comparison principle, we get $w(t)\le z(t)\le M_3=\max \{\beta _1u_0+\beta _2v_0+w_0,\frac{(1+\theta )^2}{4\theta }\left( \beta _1K_1+\beta _2K_2\right) \}$ for all $t\in (0,T_{max})$. Therefore the solution is bounded and hence $T_{max}=\infty$. Given the above analysis, (A1) and (A2) follow immediately. The proof is completed. $\square$

Now we consider the global stability of the equilibria of the system (1.2). Before proceeding, for $t>0$ and a given equilibrium $E_s=(u_s,v_s,w_s)$, we let

A3 $$\begin{aligned} \mathcal E(t;E_s):= \Gamma _1 \left( u-u_s-u_s\ln \frac{u}{u_s}\right) +\Gamma _2 \left( v-v_s-v_s\ln \frac{v}{v_s}\right) + \left( w-w_s-w_s\ln \frac{w}{w_s}\right) , \end{aligned}$$

where the constants $\Gamma _1$ and $\Gamma _2$ are given by

A4 $$\begin{aligned} \Gamma _i:= {\left\{ \begin{array}{ll} \frac{\beta _i f_i(u_s)}{\alpha _i u_s} =\beta _i,\quad & \text {if }(1.3)\text { holds},\\ \frac{\beta _i f_i(u_s)}{\gamma _i u_s} =\frac{\beta _i}{1+\gamma _ih_iu_s},\quad & \text {if }(1.4)\text { holds}, \end{array}\right. } \qquad i=1,2. \end{aligned}$$

Then we prove the global stability of the equilibria.

### A.1. Global stability for $\theta \ge L$

The first result asserts that the equilibrium $E_{uv}$ is globally asymptotically stable if $\theta \ge L$.

#### Lemma A.2

Let $\theta \ge L$, and let $f_1(u)$ and $f_2(v)$ be given by (1.3) or (1.4). Then the equilibrium $E_{uv}$ is globally asymptotically stable.

#### Proof

We first recall that $u,v,w\ge 0$ for all $t\ge 0$. Let $E_s=E_{uv}=(K_1,K_2,0)$ in (A3) and (A4). Then

$$\begin{aligned} \mathcal E(t;E_{uv})=\Gamma _1\left( u-K_1-K_1\ln \frac{u}{K_1}\right) +\Gamma _2 \left( v-K_2-K_2\ln \frac{v}{K_2}\right) +w, \end{aligned}$$

and

A5 $$\begin{aligned} \mathcal E' (t;E_{uv})&= ~\Gamma _1 \left( 1-\frac{u}{K_1}-\frac{wf_1(u)}{u}\right) \left( u-K_1\right) +\Gamma _2 \left( 1-\frac{v}{K_2}-\frac{wf_2(v)}{v}\right) \left( v-K_2\right) \nonumber \\&\quad +w\left( \beta _1 f_1(u)+\beta _2 f_2(v)-\theta \right) . \end{aligned}$$

We claim that

A6 $$\begin{aligned} \left\{ \begin{array}{l} \mathcal E(t;E_{uv})>0\text { for all }(u,v,w)\ne E_{uv},\\ \mathcal E'(t;E_{uv})\le 0{,\text { where } ``=''}\text { {holds} if and only if } (u,v,w)=E_{uv}. \end{array} \right. \end{aligned}$$

Indeed, for any given $c_0>0$, the function $\phi _1(s):=s-c_0-c_0\ln \frac{s}{c_0}$ for $s>0$ satisfies $\phi '_1(s)=1-\frac{c_0}{s}$ and $\phi ''_1(s)=\frac{c_0}{s^2}>0$, which implies that $\phi _1(s)\ge \phi _1(c_0)=0$ and $\phi _1(s)=0$ if and only if $s=c_0$. Therefore, the first conclusion in (A6) follows. Moreover, if (1.3) holds, then (A4) gives $\Gamma _1=\beta _1$ and $\beta _1 \left( f_1(u)-f_1(K_1)\right) =\Gamma _1 \frac{f_1(u)}{u}\left( u-K_1\right)$. If (1.4) holds, then (A4) gives $\Gamma _1=\frac{\beta _1}{1+\gamma _1h_1K_1}$ and

A7 $$\begin{aligned} \beta _1 \left( f_1(u)-f_1(K_1)\right) = \frac{\gamma _1\beta _1(u-K_1)}{(1+\gamma _1h_1K_1)(1+\gamma _1h_1 u)} =\Gamma _1 \frac{f_1(u)}{u}\left( u-K_1\right) . \end{aligned}$$

Similarly, we have

A8 $$\begin{aligned} \beta _2 \left( f_2(v)-f_2(K_2)\right) =\Gamma _2 \frac{f_2(v)}{v}\left( v-K_2\right) . \end{aligned}$$

Using (1.3), (1.4), (A3), (A7), (A8) and $\theta \ge L$, we have

$$\begin{aligned} w\left( \beta _1 f_1(u)+\beta _2 f_2(v)-\theta \right)&\le ~ \beta _1 w\left( f_1(u)-f_1(K_1)\right) +\beta _2 w \left( f_2(v)-f_2(K_2)\right) \\&= ~\Gamma _1 \frac{wf_1(u)}{u}(u-K_1)+\Gamma _2 \frac{wf_2(v)}{v}(v-K_2), \end{aligned}$$

which along with (A5) yields

$$\begin{aligned} \mathcal E'(t;E_{uv})\le -\frac{\Gamma _1}{K_1} (u-K_1)^2-\frac{\Gamma _2}{K_2} (v-K_2)^2. \end{aligned}$$

The above inequality indicates that $\mathcal E'(t;E_{uv})\le 0$, where “=” holds in the case of $(u,v)= (K_1,K_2)$. Note that if $(u,v)=(K_1,K_2)$, the first equation of (1.2) becomes $0=wf_1(K_1)$, which implies $w=0$ due to $f_1(K_1)>0$. Therefore, $\mathcal E'(t;E_{uv})<0$ if $(u,v,w)\ne E_{uv}$. Clearly, (A5) implies $\mathcal E'(t;E_{uv})=0$ for $(u,v,w)= E_{uv}$. Hence (A6) is proved. With (A6) and LaSalle’s invariant principle (cf. (LaSalle 1960, Theorem 3)), the proof is completed. $\square$

In what follows we assume $\theta \in (0, L)$ and consider two types of functional responses separately.

### A.2. Global stability for $\theta \in (0,L)$ and Holling type I $(1.3)$

We next show that the unique coexistence equilibrium $P_*$ is globally asymptotically stable as long as it exists.

#### Lemma A.3

(Global stability of $P_*$). Let (1.3) hold and $\theta \in (\theta _0,L )$. Then the unique coexistence equilibrium $P_*=\left( u_*,v_*,w_*\right)$ of (1.2) is globally asymptotically stable.

#### Proof

Let $E_s=P_*=\left( u_*,v_*,w_*\right)$ in (A3) and (A4). Then (A4) implies $\Gamma _i=\beta _i$, $i=1,2$. Using (1.2), (1.3), (A3), (A4) and the fact that

$$\begin{aligned} \theta =\beta _1f_1(u_*)+\beta _2f_2(v_*)=\alpha _1\beta _1 u_*+\alpha _2\beta _2 v_*,\quad 1=\frac{u_*}{K_1}+\alpha _1w_* =\frac{v_*}{K_2}+\alpha _2w_*, \end{aligned}$$

we have

$$\begin{aligned} \mathcal E'(t;P_*)&=~ \beta _1 \left( 1-\frac{u}{K_1}-\alpha _1 w\right) \left( u-u_*\right) +\beta _2 \left( 1-\frac{v}{K_2}-\alpha _2 w\right) \left( v-v_*\right) \\&\quad + (\alpha _1\beta _1 u+\alpha _2\beta _2 v-\theta )(w-w_*)\nonumber \\&=~\beta _1 \left( 1-\frac{u}{K_1}-\alpha _1w_*\right) \left( u-u_*\right) +\beta _2 \left( 1-\frac{v}{K_2}-\alpha _2w_*\right) \left( v-v_*\right) \\&=-\frac{\beta _1}{K_1} \left( u-u_* \right) ^2-\frac{\beta _2}{K_2} \left( v-v_* \right) ^2. \end{aligned}$$

Hence $\mathcal E'(t;P_*)\le 0$, where “=” possibly holds in the case of $(u,v)= (u_*,v_*)$. Note that if $(u,v)= (u_*,v_*)$, then $w=w_*$ since the system (1.2) admits the unique coexistence equilibrium $P_*$ for $\theta \in (\theta _0,L )$. Therefore, $\mathcal E'(t;P_*)<0$ if $(u,v,w)\ne E_{uv}$. If $(u,v,w)=P_*$, then (A3) obviously shows that $\mathcal E(t;P_*)=0$ for all $t>0$, which implies that $\mathcal E'(t;P_*)=0$ for all $t>0$. We obtain

$$\begin{aligned} \mathcal E'(t;E_{uv})\le 0,\text { where } \text {``='' holds if and only if } (u,v,w)=E_{uv}. \end{aligned}$$

Moreover, the same arguments as in the proof of Lemma A.2 yield that $\mathcal E(t;P_*)>0$ for $(u,v,w)\ne P_*$. Then the proof is completed by an application of LaSalle’s invariant principle. $\square$

Note that $\theta _0=0$ if and only if $\alpha _1=\alpha _2$. If $\alpha _1=\alpha _2$, then Lemma A.2 and Lemma A.3 imply that for any $\theta >0$, either $E_{uv}$ or $P_*$ is globally asymptotically stable. We next consider the case $\alpha _1\ne \alpha _2$, which implies $\theta _0>0$. Then in view of Table 1, the semi-coexistence equilibria $P_1$ and $P_2$ exist for $\theta \in (0,\theta _0]$ since $0<\theta _0<\min \left\{ L_1,L_2\right\}$. The following result gives the global stability of $P_1$ and $P_2$.

#### Lemma A.4

(Global stability of $P_1$ and $P_2$). Let (1.3) hold, $\alpha _2>\alpha _1$ (resp. $\alpha _1>\alpha _2$) and $\theta \in (0,\theta _0]$. Then the semi-coexistence equilibrium $P_1$ (resp. $P_2$) of (1.2) is globally asymptotically stable.

#### Proof

Without loss of generality, we only prove the global stability for $P_1=\left( u_{P_1},0,w_{P_1}\right) =(\frac{\theta }{\alpha _1\beta _1}, 0,\frac{L_1-\theta }{\alpha _1L_1})$ in the case of $\alpha _2>\alpha _1$, and the proof for $P_2$ in the case of $\alpha _1>\alpha _2$ is similar. Let $E_s=P_1$ in (A3) and (A4), then (A4) implies $\Gamma _i=\beta _i$, $i=1,2$. Clearly, $0<\theta _0=(1-\frac{\alpha _1}{\alpha _2})L_1<L_1$, and hence

$$\begin{aligned} \alpha _2 {w_{P_1}}=\frac{\alpha _2}{\alpha _1}\left( 1-\frac{\theta }{L_1}\right) \ge \frac{\alpha _2}{\alpha _1}\left( 1-\frac{\theta _0}{L_1}\right) = 1, \end{aligned}$$

which alongside (1.2), (1.3), $\theta =\alpha _1\beta _1 u_{P_1}$ and $\alpha _1 w_{P_1}=1-\frac{u_{P_1}}{K_1}$ implies that

$$\begin{aligned} \mathcal E'(t;P_1)&= ~\beta _1 \left( 1-\frac{u}{K_1}-\alpha _1w\right) \left( u-u_{P_1}\right) +\beta _2 \left( 1-\frac{v}{K_2}-\alpha _2 w\right) v\\&+\left( \beta _1 f_1(u)+\beta _2 f_2(v)-\theta \right) \left( w-w_{P_1}\right) \\&=~ \beta _1 \left( 1-\frac{u}{K_1}-\alpha _1{w_{P_1}}\right) \left( u-u_{P_1}\right) +\beta _2 \left( 1-\frac{v}{K_2}-\alpha _2 {w_{P_1}}\right) v\\&\le -\frac{\beta _1}{K_1}\left( u-u_{P_1}\right) ^2 -\frac{\beta _2}{K_2}v^2. \end{aligned}$$

The rest of the proof is similar to that of Lemma A.2, and we omit it for brevity. $\square$

### A.3. Global stability for $\theta \in (0,L )$ and Holling type II $(1.4)$

We now consider the case of Holling type II (1.4). We first give the global stability of semi-coexistence equilibria $Q_1$ and $Q_2$.

#### Lemma A.5

(Global stability of $Q_1$ and $Q_2$). Let (1.4) hold and $\theta \in (0,L_1)$ (resp. $\theta \in (0,L_2)$). Then the semi-coexistence equilibrium $Q_1$ (resp. $Q_2$) of (1.2) is globally asymptotically stable if (2.1) (resp. (2.2)) holds.

#### Proof

Without loss of generality, we only prove the global stability for $Q_1=\left( u_{Q_1},0,w_{Q_1}\right)$, and the case for $Q_2$ can be proved similarly. Let $E_s=Q_1=\left( u_{Q_1},0,w_{Q_1}\right)$ in (A3) and (A4). Then (A4) implies

A9 $$\begin{aligned} \Gamma _1=\frac{\beta _1}{1+\gamma _1h_1u_{Q_1}} \quad \text {and}\quad \Gamma _2=\beta _2 \end{aligned}$$

If $v_0\le K_2$, then (A1) implies $v(t)\le K_2$ for all $t\ge 0$. This along with (2.1) and the fact $\frac{f_2(s)}{s}$ decreases for $s\ge 0$ indicates that $\frac{w_{Q_1}f_2(v)}{v}\ge \frac{w_{Q_1}f_2(K_2)}{K_2}\ge 1$ for all $t\ge 0$. Similarly, if $v_0> K_2$, then (2.1) implies $\frac{K_2}{f_2(K_2)}< w_{Q_1}$. Hence (A2) yields $T_1>0$ such that $\frac{w_{Q_1}f_2(v)}{v}>1$ for all $t\ge T_1$. Therefore, for $v_0\ge 0$, it holds that

A10 $$\begin{aligned} \frac{w_{Q_1}f_2(v)}{v}\ge 1\quad \text {for all }t\ge T_1. \end{aligned}$$

Using $\theta =\beta _1f_1(u_{Q_1})$, (1.4), (A9) and (A10), we have

$$\begin{aligned} \beta _1 f_1(u)-\theta =\beta _1\left( f_1(u)-f_1(u_{Q_1})\right) =\beta _1 \frac{\gamma _1\left( u-u_{Q_1}\right) }{\left( 1+\gamma _1h_1u\right) \left( 1+\gamma _1h_1u_{Q_1}\right) }=\Gamma _1\frac{f_1(u)}{u}\left( u-u_{Q_1}\right) . \end{aligned}$$

Consequently,

$$\begin{aligned} \mathcal E'(t;Q_1)&= ~\Gamma _1 \left( 1-\frac{u}{K_1}-\frac{wf_1(u)}{u}\right) \left( u-u_{Q_1}\right) +\beta _2 \left( 1-\frac{v}{K_2}-\frac{wf_2(v)}{v}\right) v\\&+\left( \beta _1 f_1(u)+\beta _2 f_2(v)-\theta \right) \left( w-w_{Q_1}\right) \\&=~ \Gamma _1 \left( 1-\frac{u}{K_1}-\frac{w_{Q_1}f_1(u)}{u}\right) \left( u-u_{Q_1}\right) +\beta _2 \left( 1-\frac{v}{K_2}-\frac{w_{Q_1}f_2(v)}{v}\right) v\\&= -\frac{\Gamma _1h_1\gamma _1(\lambda _1+u_{Q_1}-K_1+u)}{K_1(1+h_1\gamma _1u)}(u-u_{Q_1})^2 -\frac{\beta _2}{K_2}v^2 +\beta _2 \left( 1-\frac{w_{Q_1}f_2(v)}{v}\right) v\\&\le -\frac{\Gamma _1h_1}{K_1}f_1(u)(u-u_{Q_1})^2 -\frac{\beta _2}{K_2}v^2\quad \text {for all } t\ge T_1. \end{aligned}$$

Then the global stability of $Q_1$ follows from the Lyapunov function method and LaSalle’s invariant principle, similar as in the proof of Lemma A.2. $\square$

We next prove the global stability of the coexistence equilibrium $Q_*$.

#### Lemma 4.1

(Global stability of $Q_*$). Let (1.4) hold, $\theta \in (0,L)$ and $Q_*$ be a coexistence equilibrium of (1.2). Then $Q_*$ is globally asymptotically stable if (2.3) holds.

#### Proof

Let $E_s=Q_*=\left( u_*,v_*,w_*\right)$ in (A3) and (A4). Then (A4) gives

$$\begin{aligned} \Gamma _1= \frac{\beta _1 }{1+\gamma _1h_1u_*},\quad \Gamma _2= \frac{\beta _2 }{1+\gamma _2h_2v_*}. \end{aligned}$$

Using (1.2), (1.3) and $\theta =\beta _1 f_1(u_*)+\beta _2 f_2(v_*)$, we obtain

A11 $$\begin{aligned} \mathcal E' (t;Q_*)&=~ \Gamma _1 \left( 1-\frac{u}{K_1}-\frac{wf_1(u)}{u}\right) \left( u-u_*\right) +\Gamma _2 \left( 1-\frac{v}{K_2}-\frac{wf_2(v)}{v}\right) \left( v-v_*\right) \nonumber \\&\quad + \beta _1\left( f_1(u)-f_1(u_*)\right) \left( w-w_*\right) +\beta _2\left( f_2(v)-f_2(v_*)\right) \left( w-w_*\right) . \end{aligned}$$

Similar as in driving (A7) and (A8), we have

A12 $$\begin{aligned} \beta _1\left( f_1(u)-f_1(u_*)\right) =\Gamma _1\frac{f_1(u)}{u}(u-u_*) \quad \text {and}\quad \beta _2\left( f_2(v)-f_2(v_*)\right) =\Gamma _2\frac{f_2(v)}{v}(v-v_*). \end{aligned}$$

Substituting (A12) into (A11) and using $w_*=\frac{u_*}{f_1(u_*)}(1-\frac{u_*}{K_1})=\frac{v_*}{f_2(v_*)}(1-\frac{v_*}{K_2})$ yields

$$\begin{aligned} \mathcal E'(t;Q_*)&=~ \Gamma _1 \left( 1-\frac{u}{K_1}-\frac{w_*f_1(u)}{u}\right) \left( u-u_*\right) +\Gamma _2 \left( 1-\frac{v}{K_2}-\frac{w_*f_2(v)}{v}\right) \left( v-v_*\right) \\&=-\frac{\Gamma _1h_1\gamma _1(\lambda _1+u_*-K_1+u)}{K_1(1+h_1\gamma _1u)}(u-u_*)^2- \frac{\Gamma _2 h_2\gamma _2(\lambda _2+v_*-K_2+v)}{K_2(1+h_2\gamma _2v)}(v-v_*)^2\\&\le -\frac{\Gamma _1h_1}{K_1}f_1(u)(u-u_*)^2- \frac{\Gamma _2h_2}{K_2}f_2(v)(v-v_*)^2. \end{aligned}$$

Similar arguments with the Lyapunov function method alongside LaSalle’s invariant principle as above complete the proof. $\square$

#### Proof of Theorem 2.1

In view of Lemmas A.2, A.3 and A.4, Theorem 2.1 is proved. $\square$

#### Proof of Theorem 2.2

With the results from Lemmas A.2, A.5 and 4.1, Theorem 2.2 is obtained. $\square$

## Appendix B. Proof for Remark 2.2

This appendix is dedicated to proving the conclusion stated in Remark 2.2.

### Proof for Remark 2.2

We first prove

B1 $$\begin{aligned} \Lambda _1\cap \Lambda _2=\emptyset . \end{aligned}$$

Note that $w_{Q_1}$ strictly increases with respect to $K_1>0$ since

$$\begin{aligned} \frac{d w_{Q_1}}{d K_1} =\frac{u_{Q_1}^2}{f_1(u_{Q_1}) K_1^2} =\frac{\beta _1 u_{Q_1}^2}{\theta K_1^2}>0. \end{aligned}$$

Let $(K_1, K_2)\in \Lambda _1$. If $\gamma _1\ge \gamma _2$, then the first condition in $\Lambda _1$ implies that

$$\begin{aligned} w_{Q_1}\le w_{Q_1}\big |_{K_1=\lambda _1+u_{Q_1}}=\frac{1}{\gamma _1}\le \frac{1}{\gamma _2}<\frac{1+\gamma _2h_2K_2}{\gamma _2}=\frac{K_2}{f_2(K_2)}, \end{aligned}$$

which contradicts $(K_1, K_2)\in \Lambda _1$. Therefore, $\Lambda _1=\emptyset$ in the case of $\gamma _1\ge \gamma _2$. Similarly one can show that $\Lambda _2=\emptyset$ in the case of $\gamma _1\le \gamma _2$. The claim (B1) is proved.

Without loss of generality, we next assume $\gamma _1\ge \gamma _2$, then $\Lambda _1=\emptyset$. It remains to prove that

B2 $$\begin{aligned} \Lambda _2\cap \Lambda _*=\emptyset . \end{aligned}$$

Assuming that there exists a pair $(K_1, K_2)\in \Lambda _2\cap \Lambda _*$, we shall derive a contradiction. Using

$$\begin{aligned} \theta =\beta _2f_2(v_{Q_2})=\beta _{1} f_{1}(u_*)+\beta _{2} f_{2}(v_*) \end{aligned}$$

and the fact that $f_i(s)$ and $\frac{s}{f_i(s)}$ ($i=1,2$) strictly increase with respect to $s\ge 0$, we have

B3 $$\begin{aligned} v_*<v_{Q_2}<K_2 \quad \text {and}\quad w_*=\frac{u_*}{f_1(u_*)}\left( 1-\frac{u_*}{K_1}\right)<\frac{u_*}{f_1(u_*)}< \frac{K_1}{f_1(K_1)}. \end{aligned}$$

By the second condition in $\Lambda _2$ and (B3), we have $w_*<w_{Q_2}$, which means that

B4 $$\begin{aligned} w_*=\varphi _0(v_*)<\varphi _0(v_{Q_2})=w_{Q_2}, \end{aligned}$$

where

B5 $$\begin{aligned} \varphi _0(s)&:=~\frac{s}{f_2(s)}\left( 1-\frac{s}{K_2}\right) =\frac{(K_2-s) (1+h_2\gamma _2 s)}{K_2\gamma _2}\nonumber \\&=-\frac{h_2}{K_2}\left( s-\frac{K_2-\lambda _2}{2}\right) ^2+\frac{h_2(K_2-\lambda _2)^2}{4K_2}+\frac{1}{\gamma _2},\quad s\in [0,K_2]. \end{aligned}$$

The combination of the first equation of (B3), (B4) and (B5) implies that

B6 $$\begin{aligned} v_*<\frac{K_2-\lambda _2}{2} \quad \text {and}\quad v_*+v_{Q_2}<K_2-\lambda _2. \end{aligned}$$

Starting from the first condition in $\Lambda _2$, the second condition in $\Lambda _*$, and the second inequality in (B6), we obtain $2K_2<2\lambda _2+v_*+v_{Q_2}<2\lambda _2+K_2-\lambda _2$, which simplifies to $K_2\le \lambda _2$. Therefore, the first inequality in (B6) indicates $v_*\le 0$ which is absurd. This proves (B2) and hence proves that $\Lambda _1$, $\Lambda _2$ and ${\Lambda _*}$ are mutually disjoint. $\square$

## Appendix C.

This appendix is devoted to proving that the rescaled system (3.7) with (3.8) has at most one coexistence equilibrium $Q_*=( u_*, v_*, w_*)$ (see (3.9)), which exists if and only if $\theta \in (\Theta _1, L)$, where $\Theta _1$ and *L* are given by (3.10). Remark 2.1 implies $\theta \in (0,L)$ is a necessary condition for the existence of $Q_*$. Therefore, we shall consider $\theta \in (0, L)$ below. Within this appendix, we shall use the notations defined in (3.10). For clarity, we also introduce the following notations.

- For $\gamma _2\in (0,1)$, $b>0$ and $\theta \in (0,L)$, let
  C1 $$\begin{aligned} v_M:=\frac{\theta }{\gamma _2 (b-\theta )} \end{aligned}$$
  be a positive constant (note that (3.10) implies $\theta <b$), then $v_M$ strictly increases in $\theta \in (0,L)$ and
  C2 $$\begin{aligned} v_M {\left\{ \begin{array}{ll} <1,\quad & \text {if }\theta \in (0,L_2),\\ \ge 1,\quad & \text {if }\theta \in [L_2,L). \end{array}\right. } \end{aligned}$$
- It is straightforward to check that either of the equations
  $$\begin{aligned} {\left\{ \begin{array}{ll} 8 s^3+7 s^2-8 s+1=0,\\ 24 s^3-13 s^2-6 s+3=0, \end{array}\right. } \quad s\in \mathbb {R}, \end{aligned}$$
  has two positive (real) roots and one negative (real) root. Denote the two positive roots of the first equation by $\eta _1$ and $\eta _3$ with $\eta _1<\eta _3$, and the largest root of the second equation by $\eta _4$. Let $\eta _2=\frac{4 \sqrt{7}-7}{9}$. Then
  $$\begin{aligned} (\eta _1,\eta _2,\eta _3,\eta _4)\approx (0.1471,0.3981,0.5429,0.6195). \end{aligned}$$
- For $b>0$, define the functions
  $$\begin{aligned} \xi _1(\gamma _2):=&\frac{b \left( 4 \gamma _2^2-5 \gamma _2+1\right) }{1-3 \gamma _2},\quad \gamma _2\in (\eta _1,\frac{1}{4})\cup (\eta _3,1),\\ \xi _2(\gamma _2):=&\frac{b}{3} (4-\gamma _2-\sqrt{\gamma _2^2+\gamma _2+1}),\quad \gamma _2\in (\eta _2,1). \end{aligned}$$
  It holds that $\xi _1(\gamma _2)$ strictly decreases in each connected domain with $\xi _1(\gamma _2)\in (0,L)$,
  $$\begin{aligned} \lim _{\gamma _2\rightarrow \eta _1}\xi _1(\gamma _2)= \lim _{\gamma _2\rightarrow \eta _3}\xi _1(\gamma _2)=L \quad \text {and}\quad \lim _{\gamma _2\rightarrow \frac{1}{4}}\xi _1(\gamma _2)= \lim _{\gamma _2\rightarrow 1}\xi _1(\gamma _2)=0. \end{aligned}$$
  The function $\xi _2(\gamma _2)$ strictly decreases in $(\eta _2,1)$ with $\xi _2(\gamma _2)\in (m_b,L)$,
  $$\begin{aligned} \lim _{\gamma _2\rightarrow \eta _2}\xi _2(\gamma _2)=L \quad \text {and}\quad \lim _{\gamma _2\rightarrow 1}\xi _2(\gamma _2)=m_b, \end{aligned}$$
  where $m_b:=(1-\frac{1}{\sqrt{3}})b$. Moreover, for $\gamma _2\in (\eta _3,1)$,
  $$\begin{aligned} {\left\{ \begin{array}{ll} \xi _1(\gamma _2)>\xi _2(\gamma _2),\quad & \text {if }\gamma _2\in (\eta _3,\eta _4),\\ \xi _1(\eta _4)=\xi _2(\eta _4)\approx 0.6550,\\ \xi _1(\gamma _2)<\xi _2(\gamma _2),\quad & \text {if }\gamma _2\in (\eta _4,1). \end{array}\right. } \end{aligned}$$
  The graphs of three functions $\frac{\xi _1(\gamma _2)}{b}$, $\frac{\xi _2(\gamma _2)}{b}$ and $\frac{L}{b}=\frac{1+3\gamma _2}{2(1+\gamma _2)}$ are shown in Fig. 9(a)
- For $b>0$, $\gamma _2\in (0,1)$ and $0<\theta <L$, let
  C3 $$\begin{aligned} G(s):=\sum _{k=0}^4 D_k s^k,\quad s\ge 0, \end{aligned}$$
  where the coefficients are given by
  C4 $$\begin{aligned} {\left\{ \begin{array}{ll} D_4=\gamma _2^3 ( 2 b-\theta )^2,\\ D_3=\gamma _2^2 (2 b-\theta ) (2 b (2-\gamma _2)-\theta (3-\gamma _2)),\\ D_2=(\gamma _2-1) \gamma _2 \left( b^2 (3 \gamma _2-5)-2 b \theta (\gamma _2-4)-3 \theta ^2\right) ,\\ D_1=b^2 \left( 4 \gamma _2^2-5 \gamma _2+1\right) -2 b \theta \left( 2 \gamma _2^2-4 \gamma _2+1\right) +\theta ^2 (1-3 \gamma _2),\\ D_0=b^2 (\gamma _2-1)-2 b \theta (\gamma _2-1)-\theta ^2. \end{array}\right. } \end{aligned}$$

By elementary analysis (omitted for brevity), we have the following result concerning the signs of the coefficients given in (C4).

### Proposition C.1

Let $b>0$, $\gamma _2\in (0,1)$ and $\theta \in (0,L)$. Then $D_4,D_3>0$, $D_0<0$, and

$$\begin{aligned} D_1 {\left\{ \begin{array}{ll} <0,\quad & \text {if } (\gamma _2,\theta ) \in \sum _1^-:=(\eta _1,\frac{1}{4}]\times (\xi _1,L)\cup (\frac{1}{4},\eta _3]\times (0,L)\cup (\eta _3,1)\times (0,\xi _1),\\ =0,\quad & \text {if }(\gamma _2,\theta )\in \sum _1^0:=(\eta _1,\frac{1}{4})\times \{\xi _1(\gamma _2)\}\cup (\eta _3,1)\times \{\xi _1(\gamma _2)\},\\ >0,\quad & \text {if } (\gamma _2,\theta ) \in \sum _1^+:=(0,\eta _1]\times (0,L) \cup (\eta _1,\frac{1}{4})\times (0,\xi _1)\cup (\eta _3,1)\times (\xi _1,L), \end{array}\right. } \end{aligned}$$

and

$$\begin{aligned} D_2 {\left\{ \begin{array}{ll} <0,\quad & \text {if } (\gamma _2,\theta ) \in \sum _2^-:=(\eta _2,1)\times (\xi _2,L),\\ =0,\quad & \text {if }(\gamma _2,\theta ) \in \sum _2^0:=(\eta _2,1)\times \{\xi _2(\gamma _2)\},\\ >0,\quad & \text {if } (\gamma _2,\theta ) \in \sum _2^+:=(0,\eta _2]\times (0,L) \cup (\eta _2,1)\times (0,\xi _2). \end{array}\right. } \end{aligned}$$

Proposition C.1 provides a geometric illustration for the signs of $D_1$ and $D_2$ in the $\gamma _2$-$\theta /b$ plane within $(\gamma _2,\theta )\in (0,1)\times (0,L)$, as shown in Fig. 9(b)-(c). Based on Proposition C.1, we get the following results with tedious but elementary calculations.

### Proposition C.2

Let $b>0$, $\gamma _2\in (0,1)$ and $\theta \in (0,L)$. Then the function *G*(*s*) defined by (C3) has exactly one real root in $(0,+\infty )$.

We can now prove the main result of this appendix.

### Lemma C.3

The rescaled system (3.7) with (3.8) has a unique coexistence equilibrium $Q_*=( u_*, v_*, w_*)$ if and only if $\theta \in (\Theta _1, L)$. Moreover,

$$\begin{aligned} u_* =\frac{\gamma _2 v_* (b-\theta )(v_M- v_*)}{\gamma _2 v_* (2 b-\theta )+b-\theta },\quad w_*=(1- v_* ) \left( v_* +\frac{1}{\gamma }_2\right) , \end{aligned}$$

and $v_*\in (0,\min \left\{ 1,v_M\right\} )$ satisfies $G(v_*)=0$, where the positive constant $v_M$ and the function *G* are given by (C1) and (C3), respectively.

### Proof

Clearly, it follows from (C3) and Proposition C.1 that $G(0)=D_0<0$, which alongside Proposition C.2 implies that the rescaled system (3.7) with (3.8) has a unique coexistence equilibrium $Q_*=( u_*, v_*, w_*)$ if and only if $G(\min \left\{ 1,v_M\right\} ) >0$. If $\theta \in [L_2,L)$, then (C2) implies $G(\min \left\{ 1,v_M\right\} )=G(1)=2b \gamma _2 (\gamma _2+1)^2 (L-\theta )>0$. If $\theta \in (0,L_2)$, then

$$\begin{aligned} G(\min \left\{ 1,v_M\right\} )= G(v_M) = \frac{b^4 \varphi _2(\theta )}{\gamma _2 (b-\theta )^4}, \end{aligned}$$

where $\varphi _2(\theta )$ is given by (3.11), and $\varphi _2(\theta )>0$ if and only if $\theta >\Theta _1$. The proof is completed. $\square$
